# RADseq dataset with 90% missing data fully resolves recent radiation of *Petalidium* (Acanthaceae) in the ultra‐arid deserts of Namibia

**DOI:** 10.1002/ece3.3274

**Published:** 2017-08-30

**Authors:** Erin A. Tripp, Yi‐Hsin Erica Tsai, Yongbin Zhuang, Kyle G. Dexter

**Affiliations:** ^1^ Department of Ecology & Evolutionary Biology UCB 334 University of Colorado Boulder CO USA; ^2^ Museum of Natural History UCB 350 University of Colorado Boulder CO USA; ^3^ School of GeoSciences University of Edinburgh Edinburgh UK; ^4^ Royal Botanic Garden Edinburgh Edinburgh UK

**Keywords:** de novo assembly, desert, RADseq, reference‐based assembly, speciation, stacks

## Abstract

Deserts, even those at tropical latitudes, often have strikingly low levels of plant diversity, particularly within genera. One remarkable exception to this pattern is the genus *Petalidium* (Acanthaceae), in which 37 of 40 named species occupy one of the driest environments on Earth, the Namib Desert of Namibia and neighboring Angola. To contribute to understanding this enigmatic diversity, we generated RADseq data for 47 accessions of *Petalidium* representing 22 species. We explored the impacts of 18 different combinations of assembly parameters in de novo assembly of the data across nine levels of missing data plus a best practice assembly using a reference Acanthaceae genome for a total of 171 sequence datasets assembled. RADseq data assembled at several thresholds of missing data, including 90% missing data, yielded phylogenetic hypotheses of *Petalidium* that were confidently and nearly fully resolved, which is notable given that divergence time analyses suggest a crown age for African species of 3.6–1.4 Ma. De novo assembly of our data yielded the most strongly supported and well‐resolved topologies; in contrast, reference‐based assembly performed poorly, perhaps due in part to moderate phylogenetic divergence between the reference genome, *Ruellia speciosa*, and the ingroup. Overall, we found that *Petalidium*, despite the harshness of the environment in which species occur, shows a net diversification rate (0.8–2.1 species per my) on par with those of diverse genera in tropical, Mediterranean, and alpine environments.

## 1 INTRODUCTION

Per unit geographic area, wet tropical regions such as the Amazon Basin, and Mediterranean ecosystems such as the Cape and California Floristic Provinces, host the highest levels of plant species diversity worldwide (Ackerly, 2009; Bass et al., 2010; Klak, Reeves, & Hedderson, 2004; Martínez‐Cabrera, Schlichting, Silander, & Jones, 2012). Correlates of high diversity in these regions include several abiotic factors such as time, geographic space, ample precipitation, and high temperature, as well as biotic factors such as competitive interactions and density and diversity of symbioses (Dobzhansky, 1950; Ehrlich & Raven, 1964; Mittelbach et al., 2007; Rull, 2011; Stebbins, 1970; Tripp & McDade, 2013; Tripp & Tsai, 2017). In contrast, deserts often have strikingly low levels of plant diversity, particularly infrageneric diversity, even those found in tropical latitudes (Heibl & Renner, 2012; Rundel et al., 1991). One example of this is the flora of the Atacama that, while highly endemic, has very few genera that contain >10 species (Rundel et al., 1991). Although we are unaware of any explicit hypotheses or tests to have addressed this discrepancy, a combination of variables ranging from lower organismal densities to harsher climatic regimes to historical factors likely contributes to the difference. Empirical data from lineages that represent marked deviations from the above pattern are needed to explain discrepancies in standing diversities between wet tropical and Mediterranean versus desert ecosystems.

**Figure 1:**
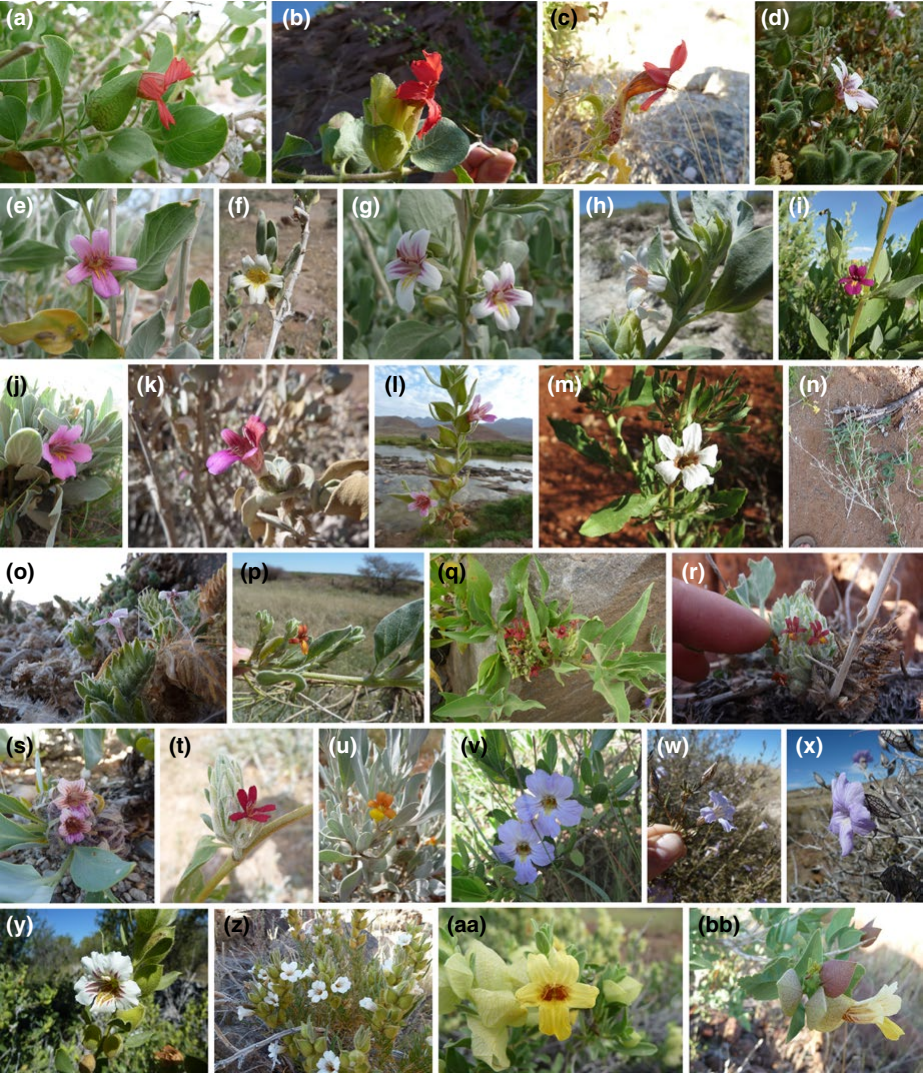
Phenotypic diversity within *Petalidium*. Images are loosely arranged phylogenetically (reflecting relationships in Figure 7 as well as predicted relationships of unsampled taxa), with upper portion of Figure 1 corresponding to upper portion of tree in Figure 7 (and vice versa for bottom portions). (a) *Petalidium coccineum* (*Tripp & Dexter 872*). (b) *Petalidium bracteatum* (*Tripp, Dexter, & McDade 4054*). (c) *Petalidium crispum* (*Tripp & Dexter* 2005). (d) *Petalidium subscrispm* (*Tripp & Dexter* 2013). (e) *Petalidium variabile* (*Tripp & Dexter 836*). (f) *Petalidium variabile* (*Tripp & Dexter 874*). (g) *Petalidium variabile* (*Tripp & Dexter 832*). (h) *Petalidium rossmannianum* (*Tripp, Dexter, & McDade 4053*). (i) *Petalidium* sp. (*Tripp, Dexter, & McDade 4075*). (j) *Petalidium ohopohense* (*Tripp & Dexter 849*). (k) *Petalidium pilosi‐bracteolatum* (*Tripp & Dexter 4096*). (l) *Petalidium welwitschii* (*Tripp & Dexter 4085*). (m) *Petalidium aromaticum* (*Dexter & Niemandt 6861*). (n) *Petalidium cirrhiferum* (plant without reproductive structures [yellow flower in upper left corner belongs to a different species of Acanthaceae]; *Tripp, Dexter, & McDade* 4060). (o) *Petalidium angustitibum* (*Nanyeni, Tripp, Klaassen* et al. *862*). (p) *Petalidium ramulosum* (*Tripp & Dexter 4120*). (q) *Petalidium setosum* (*Tripp & Dexter 887*). (r) *Petalidium lanatum* (*Tripp & Dexter 879*). (s) *Petalidium canescens* (*Tripp & Dexter 4100*). (t) *Petalidium halimoides* (*Tripp & Dexter 833*). (u) *Petalidium engleranum* (*Tripp & Dexter 778*). (v) *Petalidium oblongifolium* (*Dexter & Niemandt 6859*). (w) *Petalidium linifolium* (*Tripp, Dexter, Nanyeni, & Hasheela 2031*). (x) *Petalidium lucens* (*Tripp, Dexter, Nanyeni, & Hasheela 2065*). (y) *Petalidium rautanenii* (*Tripp* et al. *4796*). (z) *Petalidium cymbiforme* (*Tripp, Dexter, Nanyeni, & Hasheela 2078*). (aa) *Petalidium giessii* (*Tripp & Dexter 825*). (bb) *Petalidium luteo‐album* (*Tripp & Dexter 830*). Collections are deposited at WIND and duplicated at RSA, COLO, K, E, and CAS

**Figure 2:**
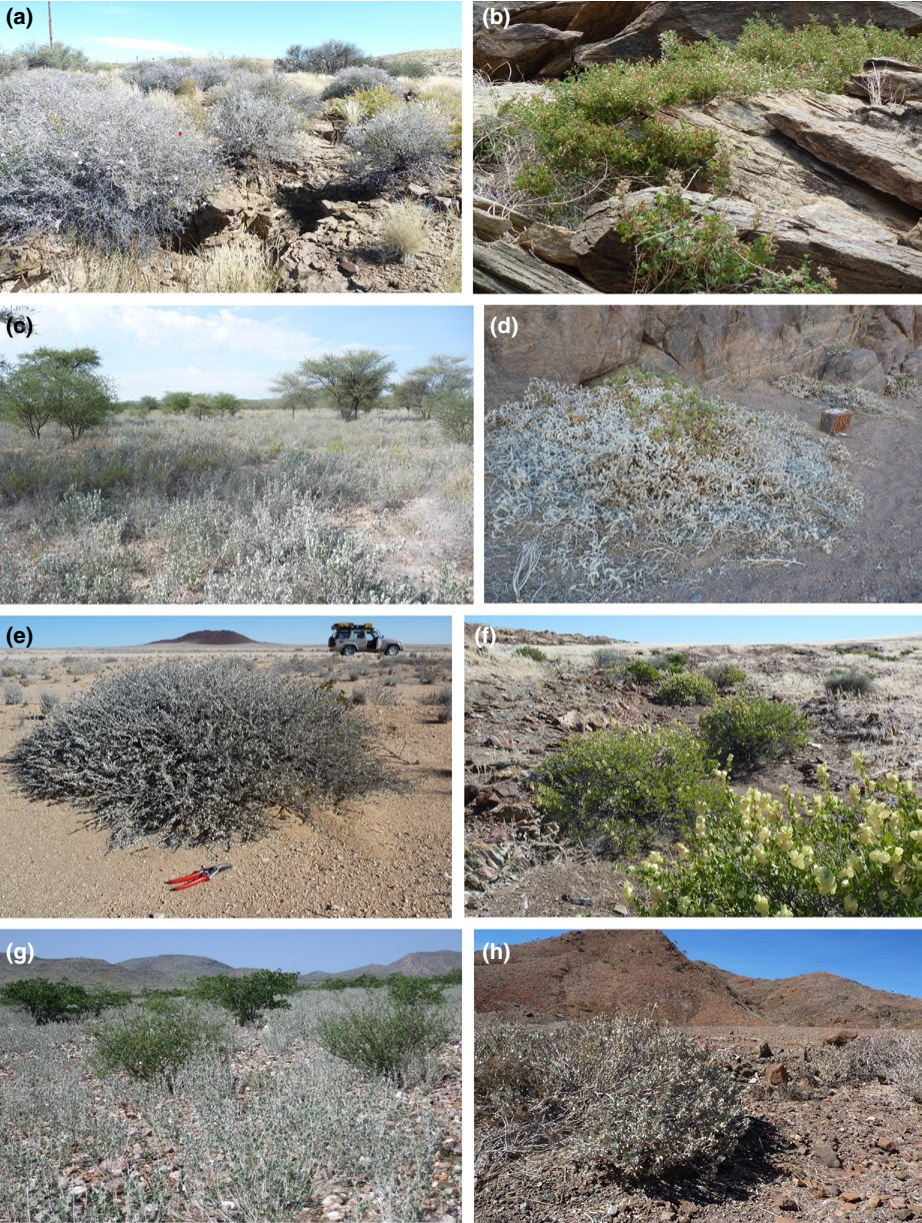
Habitat and abundance of species of *Petalidium* in Namibia. (a) *Petalidium lucens*, restricted and endemic to desert washes in southern Namibia. (b) *Petalidium crispum*, abundant in the Marienflüss, Kaokoveld. (c) *Petalidium engleranum*, one of the most dominant plants of the western Kalahari Desert. (d) *Petalidium angustitibum*, restricted yet abundant in the Hoanib River drainage. (e) *Petalidium variabile*, one of the only species of plant growing in this stretch of the Skeleton Coast. (f) *Petalidium giessii*, narrowly endemic to desert washes of Ugab River Valley to the Grootberg Mountains. (g) *Petalidium welwitschii*, the most dominant shrub in the upper Kaokoveld, for example, here in Hartmann's Valley. (h) *Petalidium variabile*, one of the most dominant shrubs of central to northwestern Namibia, here seen near the Anabeb Conservancy

**Figure 3:**
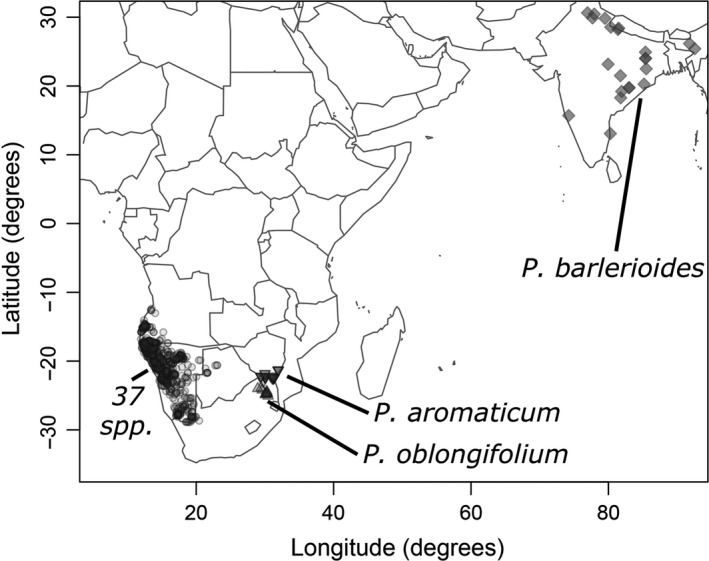
Distribution map for collections of *Petalidium*. Locations derive from a curated database (Dexter & Tripp, unpubl. data) derived from our collections and those seen by us at WIND, PRE, K, and BM. Angolan occurrences are almost certainly underrepresented in this database. Specific locations pertaining to *P. barlerioides* (diamonds), *P. aromaticum* (upside‐down triangles), and *P. oblongifolium* (rightside‐up triangles) are indicated on the map, while those of the remaining 37 species are indicated via a single icon (circles)

The flowering plant genus *Petalidium* (Acanthaceae; Figure 1) represents an extraordinary radiation concentrated in the ultra‐arid deserts of southwestern Africa but has never been studied from an evolutionary perspective beyond early taxonomic works (Meyer, 1968; Obermeijer, 1936). Except for one species in India and Nepal (*Petalidium barlerioides*), *Petalidium* is confined entirely to Namibia, Angola, and immediate surroundings (Obermeijer, 1936; Meyer, 1968; E. Tripp & K. Dexter, revision in progress). The majority of species of *Petalidium* occupy extremely small geographic ranges, several of these not exceeding 50 km^2^ (E. Tripp & K. Dexter, pers. obs.). Intriguingly, despite these narrow ranges, plants of *Petalidium* are often the dominant shrubs in the landscape (Figure 2), achieving densities unrivaled by other members of the family almost anywhere else on the planet. Species diversity is centered in the “Kaokoveld” region (Figure 3), which comprises the ultra‐arid northwestern mountains, valleys, and coast of Namibia and adjacent southwestern Angola (Craven, 2009; Gil‐Romera, Scott, Marais, & Brook, 2006), and prior authors have hypothesized this to be an “obvious area of active speciation” (Meyer, 1968; see Craven, 2009 for detailed study of the “Kaokoveld Center of Endemism”). Precipitation in the Kaokoveld is exceptionally low (<100 mm/year), falls almost entirely in the summer when evapotranspiration is extremely high, and is highly variable from year to year (Becker & Jürgens, 2000). In contrast, the nearby Nama and Succulent Karoo of South Africa and southernmost Namibia receive slightly more precipitation (100–500 mm/year) that falls in winter months and is highly consistent from year to year, thus establishing a Mediterranean climate (Cowling, Esler, & Rundel, 1999). A pattern of low infrageneric diversity in deserts seems to hold for the deserts of Namibia and Angola (Craven, 2009), aside from *Petalidium*, which represents an exceptional case of high infrageneric diversity.

That *Petalidium* may be in the early to middle stages of an evolutionary radiation challenges a paradigm of low infrageneric species richness and evolutionary diversification within deserts. To build tools to enable testing of this hypothesis as well as future investigation of drivers of speciation within the genus, we used RADseq data to generate the first phylogenetic hypotheses for *Petalidium*. Our phylogenetic investigation follows two emerging trends in the use of RADseq data: (1) that setting higher missing data thresholds for loci inclusion (i.e., allowing loci with higher levels of missing data) leads to greater phylogenetic resolution and support (Rubin, Ree, & Moreau, 2012; Wagner et al., 2013; Wessinger, Freeman, Mort, Rausher, & Hileman, 2016), and (2) that assembly of RAD loci to a reference genome should reduce the number of loci that are eventually used in phylogenetic analyses and thus reduce phylogenetic resolution and support (McCluskey & Postlethwait, 2015; Stetter & Schmid, 2017). Using a reference genome is generally thought to confer data assembly benefits by improving RADseq data quality, for example, by helping remove paralogous loci from the dataset or by correcting for sequencing error in the reads (Davey & Blaxter, 2010; Rubin et al., 2012; Hipp et al., 2014; Gonen, Bishop, & Houston, 2015; Andrews, Good, Miller, Luikart, & Hohenlohe, 2016; but see Bertels, Silander, Pachkov, Rainey, & Nimwegen, 2014). However, loci with higher rates of molecular evolution are less likely to be identified during reference genome‐based assembly and thus more likely to be excluded; these are the same loci that will have higher levels of missing data within a RADseq dataset. Thus, there likely exists a trade‐off in phylogenetic power between (1) an inclusive approach that incorporates the maximum number of loci, including those with much missing data, and (2) use of a reference genome in RADseq data assembly.

In this study, we reconstruct phylogenetic hypotheses for *Petalidium* and explore simultaneously the effects on phylogenetic resolution and support of including loci with increasing levels of missing data, of varying other parameters in RADseq data assembly, and of using a reference genome in data assembly versus de novo assembly of the data.

## 2 MATERIALS AND METHODS

### 2.1 Field methods

The large majority of confirmed species of *Petalidium* (37 of 40) occur in the Namib Desert of Namibia and Angola. Several species have expansive geographic ranges (e.g., *Petalidium variabile*, *P. welwitschii*) and are very abundant on the landscape, but others have extremely small ranges (e.g., *Petalidium subcrispum*: total distribution <35 km^2^) and are very poorly known in herbarium collections. To our knowledge, none are in formal cultivation other than in the research greenhouses of E. Tripp. To facilitate this study, we conducted four fieldtrips to Namibia and one to South Africa, with attention focused in areas of highest species diversity. Leaf tissues were preserved in silica desiccant for later molecular laboratory study. A total of 48 samples representing 22 species of *Petalidium* and one outgroup (*Barleria*, also Acanthaceae) were included in this study (Table 1). This sampling included multiple accessions of 11 species enabling assessment of RADseq data at both the species and below‐species (i.e., population) levels as well as testing of the monophyly of species of *Petalidium*. Voucher specimens of field collections were deposited at WIND, RSA, COLO, K, E, PRE, and additional institutions.

**Table 1:** The 40 accepted species of *Petalidium* (*sensu* Tripp & Dexter, this study). Species Column: in bold are the 26 species that we have seen and collected in the field; the remaining 14 species are for the most part Angolan. Representative Collections Column: voucher specimens, which are deposited at WIND with duplicates at RSA, US, CAS, COLO, K, and/or E if ours (if collections of others, location of specimen is indicated); bolded vouchers w/genetic codes indicate the specimen was used in our RADseq and/or Sanger sequencing analyses (see Figure 7 and Supporting Information). All RADseq data are deposited in GenBank as a Sequence Read Archive (Study #PRJNA392452; SRA #SRP110762). The asterisk denotes a collection that we suspect represents an as yet undescribed species, pending further study

Species	Representative collections
***Petalidium angustitibum*** **P.G. Mey.**	*Nanyeni, Tripp, Klaassen,* et al. *862*
***Petalidium aromaticum*** **Oberm.**	***Dexter & Niemandt 6861*** **(Parom)**
*Petalidium barlerioides* Nees	*Koelz 19961* **(Pbarl: US)**
***Petalidium bracteatum*** **Oberm.**	***Tripp, Dexter, & McDade 4054*** **(Pbra2)**
***Petalidium canescens*** **C.B. Clarke**	***Tripp & Dexter 882*** **(Pcan3),** ***Tripp & Dexter 4100*** **(Pcan5),** ***Tripp & Dexter 4103*** **(Pcan6),** ***Oliver*** **et al.** ***6643*** **(Pcane: MO),** ***Seydel 570*** **(Pcan2: NY)**
**Petalidium cirrhiferum S. Moore**	***Tripp, Dexter, & McDade 4060*** **(Pcir4),** ***Hearn 54*** **(Pcirr: US)**
**Petalidium coccineum S. Moore**	***Tripp & Dexter 843*** **(Pcocc),** ***Tripp & Dexter 872*** **(Pcoc2)**
**Petalidium crispum A. Meeuse ex P.G. Mey.**	***Tripp, Dexter, & McDade 4056*** **(Pcri5),** ***Tripp, Dexter, & McDade 4057*** **(Pcri4)**
**Petalidium cymbiforme Schinz**	***Tripp & Dexter 2078*** **(Pcymb)**
*Petalidium damarense* S. Moore	–
*Petalidium elatum* Benoist	–
**Petalidium engleranum C.B. Clarke**	***Tripp & Dexter 791*** **(Peng4),** ***Tripp & Dexter 816*** **(Peng5),** ***Tripp & Dexter 4051*** **(Peng6),** ***Joffe 51*** **(Peng2: US)**
**Petalidium geissii P.G. Mey.**	***Tripp & Dexter 825*** **(Pgei3),** ***Kers 252*** **(Pgies: US)**
*Petalidium glandulosum* S. Moore	–
*Petalidium glutinosum* C.B. Clarke	–
*Petalidium gossweileri* S. Moore	–
**Petalidium halimoides S. Moore**	***Tripp & Dexter 833*** **(Phali),** ***Tripp & Dexter 1965*** **(Phal4),** ***Tripp & Dexter 4077*** **(Phal2)**
*Petalidium hirsutum* (T. Anders.) P.G. Mey.	***P1972‐5174 s.n.*** **(Phirs: C)**
*Petalidium huillense* C.B. Clarke	–
**Petalidium lanatum C.B. Clarke**	***Tripp & Dexter 879*** **(Plan3),** ***Tripp & Dexter 4108*** **(Plan5),** ***Seydel 709*** **(Plana: US),** ***Seydel 709*** **(Plan4: US),** ***Seydel 4339*** **(Plan2: NY)**
*Petalidium lepidagathis* S. Moore	–
**Petalidium linifolium T. Anders.**	*Tripp, Dexter,* et al. *2031, Tripp, Dexter,* et al. *2040, Tripp, Dexter,* et al. *2084*
**Petalidium lucens Oberm.**	*Tripp, Dexter,* et al. *2065, **Brand*** **et al.** ***27*** **(Pluce: NY)**
**Petalidium luteo‐album A. Meeuse**	***Tripp & Dexter 830*** **(Plut2),** ***Tripp & Dexter 875*** **(Plut3),** ***Tripp, Dexter,*** **et al.** ***1974*** **(Plut7),** ***Tripp & Dexter 4092*** **(Plut8),** ***Tripp & Dexter 2003*** **(Plut9),** ***Smook 7713*** **(Plute: MO)**
**Petalidium oblongifolium C.B. Clarke**	***Dexter & Niemandt 6857*** **(Pobl2)**
***Petalidium ohopohense*** **P.G. Mey.**	***Tripp & Dexter 849*** **(Pohop)**
*Petalidium parvifolium* C.B. Clarke ex Schinz	***Venter & Venter 9800*** **(Pparv: NY),** ***Venter & Venter 9800*** **(Ppar2: S)**
*Petalidium physaloides* S. Moore	–
***Petalidium pilosi‐bracteolatum*** **Merxm. & Hainz**	***Tripp & Dexter 4096*** **(Ppil2),** ***Seydel 2945*** **(Ppil3: US)**
***Petalidium ramulosum*** **Schinz**	***Tripp & Dexter 4121*** ***(Pram2)***
***Petalidium rautanenii*** **Schinz**	***Tripp & Dexter 803*** ***(Prau2)*,** ***Bayliss 13019*** **(Praut: NY)**
***Petalidium rossmannianum*** **P.G. Mey.**	***Tripp & Dexter 823*** **(Pros2),** ***Tripp & Dexter 825*** **(Pros3),** ***Tripp & Dexter 840*** **(Pros4),** ***Germishuizen 1917*** **(Pross: MO)**
*Petalidium rupestre* S. Moore	–
**Petalidium setosum C.B. Clarke ex Schniz**	***Tripp & Dexter 887*** **(Pset5),** ***Tripp & Dexter 4112*** **(Pset8),** ***Seydel 569*** **(Pset3: US),** ***Seydel 2945*** **(Pset4: US)**
*Petalidium spiniferum* C.B. Clarke	–
**Petalidium subcrispum P.G. Mey.**	*Tripp, Dexter,* et al. *2013, Tripp & Dexter 4086*
*Petalidium tomentosum* S. Moore	–
**Petalidium variabile C.B. Clarke**	***Tripp & Dexter 832*** **(Pvar3),** ***Tripp & Dexter 836*** **(Pvar4),** ***Tripp & Dexter 859*** **(Pvar5),** ***Tripp & Dexter 860*** **(Pvar6),** ***Tripp & Dexter 861*** **(Pvar7),** ***Tripp & Dexter 862*** **(Pvar8),** ***Tripp & Dexter 865*** **(Pvar16),** ***Tripp & Dexter 866*** **(Pvar17),** ***Tripp & Dexter 867*** **(Pvar18),** ***Tripp & Dexter 868*** **(Pvar19),** ***Tripp & Dexter 869*** **(Pvar20),** ***Tripp & Dexter 874*** **(Pvar10),** ***Smook 7807*** **(Pvari: MO)**
**Petalidium welwitschii S. Moore**	***Tripp & Dexter 844*** **(Pwel6),** ***Tripp & Dexter 4085*** **(Pwel3),** ***Tripp & Dexter 4088*** **(Pwel4),** ***Tripp & Dexter 4091*** **(Pwel5)**
*Petalidium sp. **(cf.** ***variabile*** **)**	***Tripp, Dexter, & McDade 4075*** **(Psp8)**

### 2.2 DNA Isolation and sequencing methods

DNA was extracted from leaf tissue using either CTAB (Doyle & Doyle, 1987) or Qiagen DNEasy Plant Mini Kits. DNA sequencing proceeded via a RADseq approach (Parchman et al., 2012), which utilized a double restriction enzyme digest with *EcoR1* and *Mse1* followed by gel size selection of the 250‐ to 500‐bp region to reduce the genome sequenced. A detailed experimental protocol, based on Parchman et al.'s (2012) restriction enzyme digestion, adaptor and barcode ligation, PCR amplification, gel purification, and size selection, was consolidated into a single protocol and expanded upon (see below for custom barcode design and custom R scripts) to provide a simple, single resource for future users (Supporting Information). Following library prep, 48 pooled samples were submitted to the BioFrontiers Sequencing Facility at the University of Colorado, Boulder, for QC, column cleanup, and sequencing on an Illumina HiSeq V3 as ½ of a lane of a 1 × 100 bp single‐read direction run. Downstream analyses were conducted on the University of Colorado's JANUS supercomputer, which comprises a total of 1,368 compute nodes, each containing 12 cores, for a total of 16,416 available cores (each with 2 GB of available RAM).

### 2.3 Barcode design

In total, a set of 96 variable‐length single‐indexed barcodes were designed (Supporting Information) to facilitate pooling of up to 96 samples into a single lane. RADseq barcodes should be variable in length because the five bases sequenced immediately following them will be identical due to the nature of the cutsite of the restriction enzyme. Thus, variable‐length barcodes of 7‐ to 10‐bp stagger cutsite sequences, which in turn introduces base (frameshift) diversity into the sequencing process, drastically improving clustering during bridge amplification and base‐calling accuracy on the Illumina platform (Elshire et al., 2011).

Barcodes were designed by first creating 1,284 10‐bp constant‐length barcodes using create‐index‐sequences.py (Meyer & Kircher, 2010), with an edit distance of 4. Barcodes were then processed through a custom R script that recalculated all pairwise distances between barcodes, allowing for slippage of a single bp at the beginning or end of either barcode (Supporting Information). Barcode pairs with a minimum distance <3 bp were labeled as “possibly problematic,” and if a barcode was flagged in this manner two or more times, it was discarded. Pairwise distances were recalculated on the remaining barcode set, again allowing possible slippage of 1 bp. During this second iteration, barcodes with minimum distances <3 bp were discarded, leaving a total of 809 barcodes that remained in the working set. To design barcodes of lengths 7, 8, and 9 bp from the 10‐bp barcode set, we selected barcodes with specific end sequences that matched portions of the sequence of the *EcoRI* cutsite (CAATTC). Adaptor ligation occurs at the cutsite, and thus, the cutsite is necessarily always sequenced immediately following the barcode. As one example, for 7‐bp barcodes, we chose barcodes that had “CAA” as the last three bases, truncated the “CAA,” and what remained were barcodes that were only 7 bp long. Similarly, we designed 8‐ and 9‐bp barcodes by selecting 10‐bp barcodes with “CA” and “C” at the 3′ end positions, respectively. Twenty‐four 8‐bp and 24 9‐bp barcodes were randomly chosen to comprise the final barcode set along with all 18 7‐bp barcodes. The final 30 barcodes were chosen from the remaining 10‐bp barcodes, attempting to maximize diversity in base composition. Each site was assigned a weight based on the site's base composition, with more skewed base compositions receiving a higher weight. For each base at each site, underrepresented bases were given a higher weight. Weights were assigned to each barcode based on multiplying the weights for each base with the weights for each site. Finally, 30 10‐bp barcodes were sampled randomly based on their weighted probabilities.

### 2.4 RADSeq data processing & analyses

**Figure 4:**
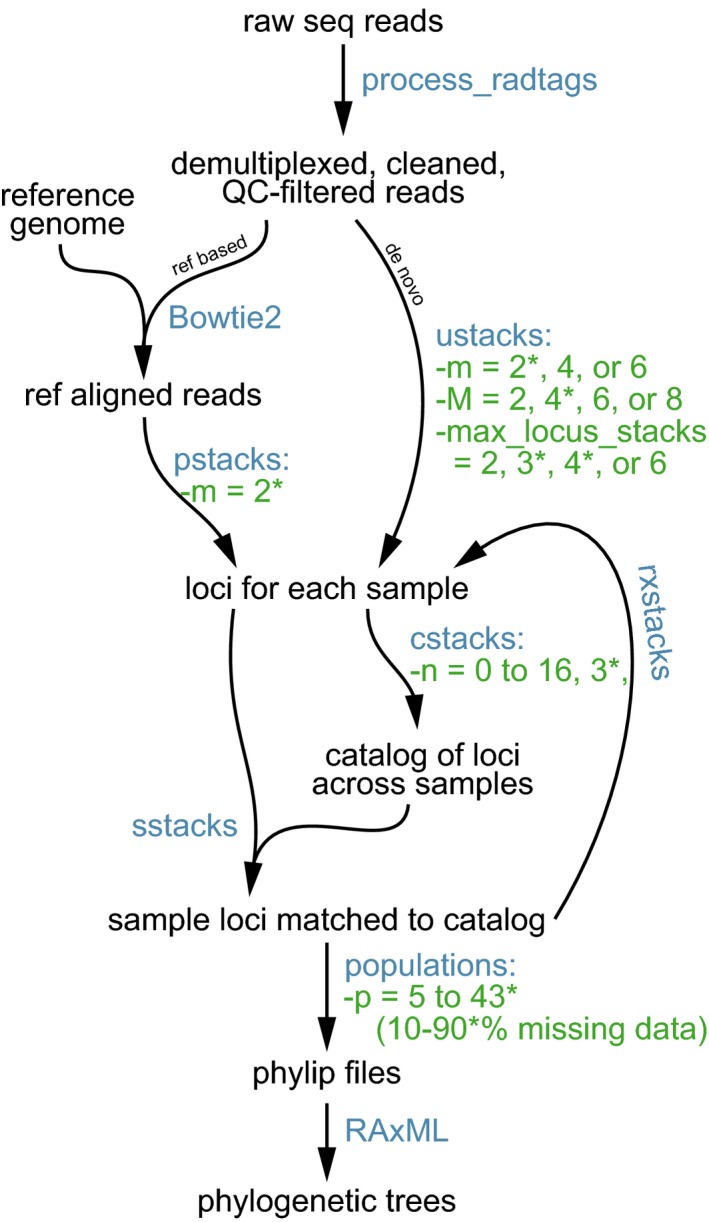
Workflow describing phylogenetic analysis of *Petalidium* plus one outgroup using RADseq data. Raw sequence reads from a 1/2 lane of Illumina HiSeq 1 × 100 bp run with 48 samples were demultiplexed, cleaned, and filtered by quality scores via the process‐radtags script. For runs 1–18, the demultiplexed reads were assembled into loci *de novo* (right branch of pathway) via ustacks, where the *m*, *M*, and *max‐locus‐stacks* parameters were assigned varying values (see Table 2 for details). For run *R*, the demultiplexed reads were first aligned to a reference genome (*Ruellia speciosa*; left branch of pathway) using Bowtie2, then assembled into loci via pstacks. The resulting stacks of loci for each sample were input into cstacks, which built a catalog of loci across all samples. The parameter *n*, implemented in cstacks, was varied from 0 to 16. Sample loci were matched to those in the catalog in the program sstacks. Loci were corrected with the program module rxstacks and rerun through cstacks and sstacks. SNPs were output to phylip files with varying levels (10%–90%) of missing data via the *p* parameter implemented in the populations script. Phylip files were input into RAxML to search for the ML tree and to conduct ML bootstrap analyses. Program inputs and outputs are in black; program names are in blue; program parameters that we varied are in green. Asterisks indicate optimal parameter values derived from our study. Process‐radtags, ustacks, pstacks, cstacks, sstacks, rxstacks, and populations are all part of the Stacks version 1.4 software package. Upstream portions of the workflow (e.g., barcode design, wet laboratory protocols) can be found in the Supporting Information

To assemble loci and generate phylip files for downstream phylogenetic analyses, raw sequence data were processed with the Stacks v. 1.40 software package (Catchen, Hohenlohe, Bassham, Amores, & Cresko, 2013), which includes the following scripts: *process-radtags*, *ustacks*, *pstacks*, *cstacks*, *sstacks*, *rxstacks*, and *populations* (Figure 4). Raw sequence reads were demultiplexed, cleaned, and filtered according to quality scores using the process‐radtags script; default parameters were assumed with a sliding window of 15 bp and phred quality score threshold of 10. Cleaned data were then assembled into loci following two main approaches: (1) de novo, that is, without a reference genome and (2) with a reference genome. For de novo assembly, 18 different combinations of four parameters were implemented: *m* (minimum stack depth), *M* (maximum between stack distance), *max‐locus‐stacks* (maximum number of stacks at a locus), and *n* (number of mismatches allowed between samples). Values of *m* ranged from 2 to 6 and control the minimum depth of coverage required for a locus stack to be initially formed; *M* ranged from 2 to 8 and controls how different two alleles can be and still be merged into the same locus; *max‐locus‐stacks* ranged from 2 to 6 and dictates how many alleles are allowed per locus; *n* ranged from 0 to 16 and controlled how different loci identified in different populations can be and still be merged into the same locus in the overall catalog. These variables are further defined in the Stacks manual (see also Wagner et al., 2013). For reference‐based assembly, we implemented a single set of parameters (Table 2) that closely mirrored the settings that yielded our best trees (i.e., most resolved and strongly supported) from de novo assembly (see 2.1). The de novo and reference‐based assemblies were implemented across nine different levels of missing data to explore resulting numbers of SNPs recovered and the impact on phylogenetic support and resolution (Table 2). In this study, we use the phrase “missing data threshold” to refer to the proportion of individuals that lack sequence data for a given locus (or the minimum number of individuals required to have sequence data at a given locus; elsewhere, this has been termed “min individuals” [Wagner et al., 2013] or “Min_taxa_” [Wessinger et al., 2016]). Thus, in this study, a 60% missing data threshold refers to a dataset in which loci were included if at least 40% of the individuals had read data for that locus.

**Table 2:** Number of SNPs recovered from each Stacks run of *Petalidium* RADseq data at multiple levels of missing data. Run 1 corresponds to default settings for *de novo* assembly in Stacks except for the *n* parameter (no default values specified by the program). Run R corresponds to the reference‐based assembly run. *m* = minimum stack depth; *M* = maximum between stack distance; mls = max‐locus‐stacks; *n *= number of mismatches allowed between sample

Run	Stacks params				% missing data								
	*m*	*M*	mls	*n*	90%	79%	71%	60%	50%	40%	29%	21%	10%
1	2	2	3	3	176,198	97,957	53,496	21,856	7,680	2,097	378	103	11
2	4	2	3	3	94,583	45,487	21,361	7,597	2,249	522	161	85	19
3	6	2	3	3	34,213	12,696	5,287	1,568	557	220	116	80	26
4	2	4	3	3	254,171	156,439	98,867	47,341	19,015	5,837	1,253	164	15
5	2	6	3	3	267,905	164,474	104,509	51,389	20,341	6,181	1,344	201	15
6	2	8	3	3	280,737	170,302	108,445	53,792	21,306	6,296	1,378	243	15
7	2	2	2	3	224,874	132,624	78,501	35,003	13,714	4,064	858	94	3
8	2	2	4	3	226,947	134,618	81,152	37,407	15,069	4,770	1,079	131	15
9	2	2	6	3	226,354	134,013	80,755	37,692	15,309	4,845	1,072	141	15
10	2	2	3	0	26,550	12,331	7,035	2,924	953	177	23	6	0
11	2	2	3	2	126,141	68,459	38,747	16,797	5,989	1,568	357	85	9
12	2	2	3	4	174,015	94,072	51,221	21,913	8,195	2,393	411	106	11
13	2	2	3	6	205,195	109,616	58,489	24,284	9,241	2,706	482	106	11
14	2	2	3	8	229,622	122,819	63,666	26,180	9,724	2,817	488	113	11
15	2	2	3	10	250,448	134,040	67,920	27,306	10,161	2,933	503	127	29
16	2	2	3	12	267,970	143,138	72,631	28,644	10,675	3,076	555	144	29
17	2	2	3	14	285,711	152,085	75,814	29,321	10,907	3,164	560	156	29
18	2	2	3	16	302,987	161,971	79,093	30,681	11,094	3,368	583	167	45
R	2	NA	NA	3	27,697	19,667	15,087	9,970	5,825	2,682	985	225	86

In the de novo approach, cleaned data were passed through the *ustacks* program, which calls loci for each individual sample. In the reference genome‐based approach, we used the “—very‐sensitive‐local” approach in Bowtie2 v. 2.2.9 (Langmead & Salzberg, 2012) and aligned the cleaned reads to the recently completed draft genome of *Ruellia speciosa* (1,021 Mb, or ~1.02 Gb; Zhuang & Tripp, 2017). *Ruellia* is in the same family and tribe as *Petalidium* (both Ruellieae: Acanthaceae; Tripp, Daniel, Fatimah, & McDade, 2013) but evolved several million years prior to *Petalidium* (crown *Ruellia*: ~10.6 Ma [Tripp & McDade, 2013] versus crown *Petalidium*: between 4.8 and 1.5 Ma [herein estimated, see 2.1]). The reference‐aligned reads were passed to the program *pstacks* with *m* set to 2, which calls loci for each sample. Whether assembled de novo or by reference, loci were then input into *cstacks*, which constructed a global catalog of loci. This loci catalog was then used by *sstacks* to match and call sample loci. The *rxstacks* corrections module was then invoked, which utilizes data from across samples to “repair” loci calls in each sample prior to re‐running *cstacks* and *sstacks*. Finally, the assembled and matched sample loci were passed to the *populations* script to create phylip files with different levels of *p* (missing data thresholds). The *populations* script can be exceptionally memory intensive, even on a high‐memory supercomputer, and as such could not be reliably run at levels of missing data higher than 50%. To circumvent this issue, the *populations* script was first run with all samples assigned to a single population and default settings. We then built a custom R script (see Supp‐Script‐creating‐whitelists.r, Supporting Information) that (1) parsed the sumstats.tsv output by the number of samples containing a given locus and (2) constructed lists of loci at varying levels of missing data thresholds (10%–90%). The *populations* program was then used to input a given list of loci, thus overcoming the memory‐intensive task of computing which loci met the missing data criterion; *populations* was then used to output most phylip files in this study. However, at the highest levels of missing data thresholds (i.e., ≥70%), *populations* was unable to output the high numbers of accompanying SNPs; as such, the loci lists at these higher missing data thresholds were further subdivided into 10 subsets each (see Supp‐Script‐creating‐whitelists.r, Supporting Information). These subset lists of loci were small enough to run successfully through the *populations* program. Output phylip files containing only informative sites (i.e., invariable sites removed) from the subset lists were then merged via a custom R script (see Supp‐Script‐cleanupphylip.r, Supporting Information). The above steps effectively splits and then recombines informative sites in a manner that facilitates the processing of high levels of SNPs in Stacks and as such does not affect downstream results in any manner.

### 2.5 Phylogenetic reconstruction

To estimate phylogenetic relationships among *Petalidium* as well as to gauge the effects of different data assembly protocols on phylogenetic reconstruction, maximum‐likelihood tree searches were conducted on the 19 runs described in Table 2, across different thresholds of missing data, using RAxML v 8.0.0 (Stamatakis, 2014). *Barleria*, which is a genus belonging to a different tribe of Acanthaceae, was used to root RADSeq trees. All analyses were conducted on a concatenated matrix utilizing a GTR + G model of sequence evolution. Support for relationships was assessed using 100 rapid bootstrap replicates. A 50% majority‐rule consensus tree was computed for each set of bootstrap trees across all analyses, and node support was visualized using a custom R script (Supp‐Script‐processing‐bootstraptrees.r, Supporting Information) that incorporated functions from the ape v. 3.2 R package (Paradis, Claude, & Strimmer, 2004). Phylogenetic support was quantified across all resulting phylogenies by tallying the proportion of nodes with bootstrap values ≥70%.

### 2.6 Divergence time estimation

To date, the origin and subsequent radiation of *Petalidium* as well as to estimate net diversification rates, we conducted divergence time analyses that benefitted from a powerful fossil dataset available for Acanthaceae (Tripp & McDade, 2014). Although no published fossil data are available for *Petalidium* specifically except for very young reports from the Holocene (Gil‐Romera et al., 2006), several fossils representative of closely related lineages in the tribe Ruellieae (sensu Tripp et al., 2013), to which *Petalidium* belongs, are available for use as primary fossil constraints. However, we generated RADseq data only for *Petalidium* plus one outgroup, both of which lack a fossil record; as such, we were unable to use our RADseq dataset for divergence time estimation. We therefore assembled a molecular matrix derived from Sanger sequencing of 44 accessions (18 species) of *Petalidium* plus seven outgroups, the latter of which are all members of Ruellieae: *Brillantaisia grottanellii*, *Duosperma longicalyx*, *Dyschoriste albiflora*, *Mellera submutica*, *Phaulopsis imbricata*, *Sanchezia speciosa*. We generated bidirectional sequences for markers that have been used in previous phylogenetic research on Acanthaceae: ITS+*5.8S*, *psbA‐trnH*, *trnG‐trnR*, and *trnG‐trnS* (Tripp, 2007, 2010; Tripp et al., 2013; information on primers and amplification conditions can be found in these publications); these markers are for the most part sufficient for yielding information on relationships among genera in Ruellieae but insufficient to resolve the phylogeny of *Petalidium* (see2.1) or other species‐level questions (e.g., within sublineages of *Ruellia*; Tripp, 2010). In total, this matrix consisted of 4,432 characters, of which 503 were parsimony informative.

Our outgroup selection allowed us to conduct two different time calibration analyses, making use of two fossils both ranked as “high utility” in Tripp & McDade (2014; see that publication for in‐depth explanation of fossil identities, clades represented, and utility assessments). These fossils were here used as minimum age constraints for different taxon sets (Table 3). Analyses conducted using BEAUTi, BEAST, Tracer, and TreeAnnotator (Drummond & Rambaut, 2007) followed methods implemented in Tripp and McDade (2014). Rate heterogeneity across branches was permitted via implementation of a relaxed clock model (Drummond, Ho, Phillips, & Rambaut, 2006), and the uncorrelated lognormal distribution was selected because previous simulation studies have demonstrated its superior performance (e.g., Drummond, Ho, Phillips, & Rambaut, 2009). A Yule speciation model was specified for the tree prior (Gernhard, Hartmann, & Steel, 2008). For each analysis, we ran BEAST for 10 million generations, which yielded sufficient sampling of posterior distributions based on resulting ESS values (Drummond & Rambaut, 2007). We discarded the first 3 million (analysis 1) or 5.1 million (analysis 2) states as burn‐in (i.e., the interval during which posterior probabilities had not yet stabilized), and the remaining trees were used to construct a 50% maximum clade credibility tree, keeping target age heights.

**Table 3:** Fossil Constraints. See Tripp and McDade (2014) for fossil number and identification, and see Tripp et al. (2013) for Ruellieae subtribe information. See Supporting Information for full set of divergence time ages and 95% credibility intervals

Parameter	Analysis 1	Analysis 2
Fossil #	36	51
Taxa Constrained	Pseudocolpate Ruellieae excluding Trichantherinae: *Brillantaisia*, *Duosperma*, *Dyschoriste*, *Mellera*, *Phaulopsis*, *Petalidium*	Petalidiinae + Mimulopsinae: *Duosperma*, *Dyschoriste*, *Mellera*, *Phaulopsis*, *Petalidium*
Age	Upper Miocene (~14.55–5.3 Ma)	Mio‐Pliocene (~23.8–1.8 Ma)
Zero Offset	5.3 Ma	1.8 Ma
Log Stdev	1.4 Ma	1.3 Ma
Mean	2.5 Ma	6.0 Ma
5% Quantile	5.4 Ma	2.1 Ma
95% Quantile	14.7 Ma	23.7 Ma

## 3 RESULTS

### 3.1 RADSeq data and loci assembly

The single lane of HiSeq 1 × 100 bp sequencing yielded 186,516,277 reads, about half of which represented our 48 *Petalidium* and one *Barleria* samples (the other half represented samples submitted by the user of the other 1/2 lane). The number of reads recovered for each sample varied substantially, ranging from only 944 reads (*Petalidium lanatum*‐3) to 662,231 reads (*Petalidium cirrhiferum*‐4; numbers after dashes refer to a specific accessions/collections of a given species). When the filtered reads were aligned to the *Ruellia speciosa* genome, the average alignment rate was 30%, with a minimum of 3% (*Petalidium lanatum*‐3) and a maximum of 51% (*Petalidium halimoides*‐2). Two samples had exceptionally few reads (*Petalidium lanatum*‐3, *Petalidium halimoides*) and as such were excluded from all downstream analyses.

### 3.2 Variation in de novo assembly strategies

**Figure 5:**
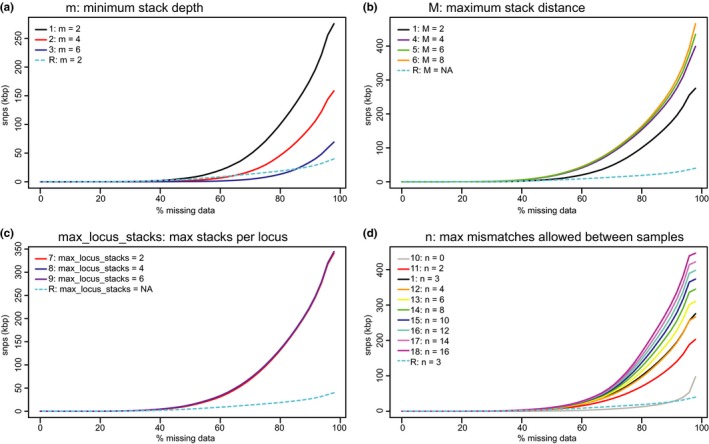
The effects of (a) minimum stack depth, (b) maximum stack distance, (c) maximum stacks allowed per locus, and (d) maximum mismatches allowed between samples on the numbers of SNPs identified in our *Petalidium* dataset. Aside from the focal parameter in each panel, all remaining parameters were at default values (see Table 2 for details)

Results from analyses indicated that altering values of *m*, *M*, *max-locus-stacks*, and *n* had a relatively minor impact on numbers of SNPs recovered, although variation in *m* yielded the largest effects: higher levels (e.g., *m *=* 4* and *m *=* 6*) yielded lower numbers of recovered SNPs (Figure 5a). Increasing *M* from 2 to 4, 6, or 8 yielded larger numbers of recovered SNPs although there were negligible differences in numbers of SNPs, as a function of the missing data threshold (Figure 5b). Whereas increasing *max-locus-stacks* had a negligible effect as a function of missing data (Figure 5c), increasing *n* from *n *=* 0* to *n *=* 2* yielded marked differences in recovered SNPs, with diminishing returns at higher values of *n* (Figure 5d).

**Figure 6:**
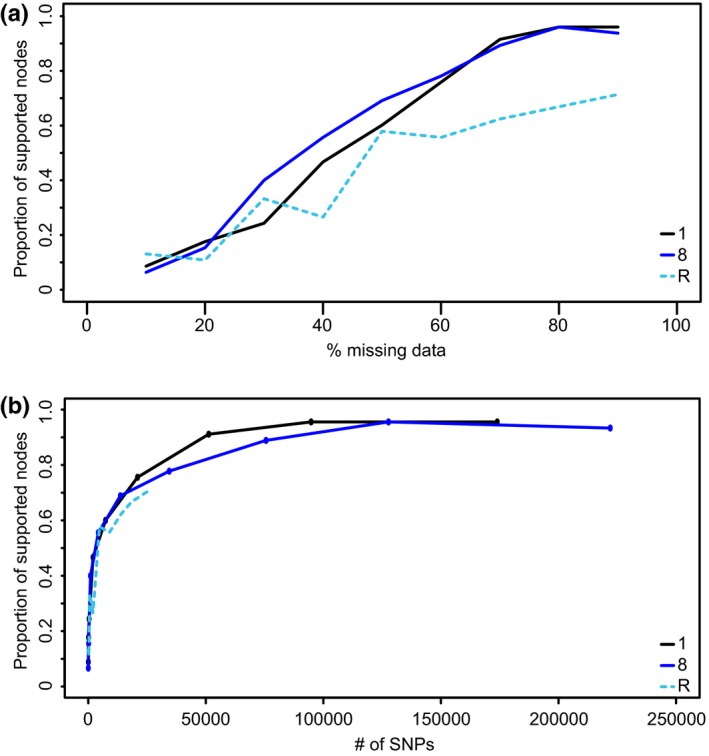
ML bootstrap trees constructed from *Petalidium* RADseq data show increased node support with increasing levels of missing data (a). Node support also follows the numbers of recovered SNPs (b). Nodes with bootstrap values ≥70% were considered to be well supported. Data from Runs 1, 8 (both *de novo* assembly), and R (reference‐based assembly) are shown

In contrast to the above, different allowances of missing data thresholds had substantial impact on the resulting numbers of SNPs, with on average a 15,000‐fold difference between our lowest missing data threshold (10%) and our highest missing data threshold (90%; Figure 6). Across all 18 combinations of the four variables plus the reference‐based assembly, increasing missing data thresholds consistently yielded increasing numbers of SNPs (Figure 6). Higher thresholds of missing data also yielded higher node support (Figure 6). Curves depicting numbers of supported nodes climbed steadily between zero and ~25,000 SNPs but began to level off after >25,000 SNPs (Figure 6). Phylogenies achieved close to their maximum number of supported nodes at 50,000 SNPs or greater, with >100,000 SNPs yielding essentially no newly supported nodes (Figure 6).

### 3.3 De novo versus reference‐based assembly

Analyses indicated that reference‐based alignment yielded slightly higher numbers of SNPs at low thresholds of missing data (primarily <30%) but de novo assembly outperformed (i.e., yielded better and more strongly resolved topologies) reference‐based assembly at higher thresholds of missing data (Figure 6). Comparison of de novo to reference‐based assembly also indicated that reference‐based assembly yielded, on the whole, topologies with fewer supported nodes across nearly all thresholds of missing data (Figure 6).

### 3.4 Phylogenetics

**Figure 7:**
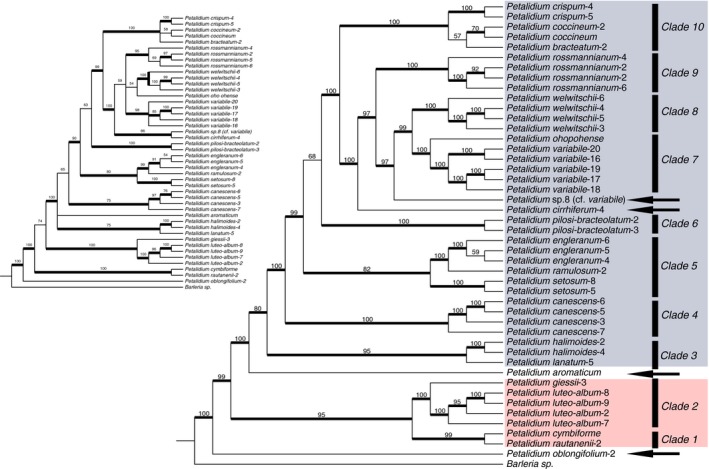
One of our best estimates of phylogenetic relationships among species of *Petalidium*. Right: results from analysis of Run 1 with 90% missing data (“R1.m90”; Table 2). The ten clades here resolved are strongly supported; all but three nodes in the phylogeny have ML bootstrap support ≥70%. Four species marked by arrows represent accessions not here assigned to clades (*P. oblongifolium*, *P. aromaticum*, *P. cirrhiferum*, and *P*. sp. 8 (vel. aff. *variabile*) but are suspected to form clades with other species following complete taxon sampling of the genus (see Table 1). Of the 11 species for which more than one accession per species was sequenced (from top to bottom: *P. crispum*, *P. coccineum*, *P. rossmannianum*, *P. welwitschii*, *P. variabile*, *P. pilosi‐bracteolatum*, *P. engleranum*, *P. setosum*, *P. canescens*, *P. halimoides*, and *P. luteo‐album*), all formed reciprocally monophyletic clades. Clades 1 & 2 (red) and clades 3–10 (blue) corroborate Obermeijer's (1936) and Meyer's (1968) classification of species into one of two sections: the first (red) composed of plants with regular, five‐parted calyces and the second (blue) composed of species with irregular, four‐parted calyces. Left (smaller inset phylogeny): results from analysis of loci assembled with a reference, with 90% missing data. Relationships are consistent with those based on the *de novo* assembly (right) but are less resolved and less well supported

**Figure 8:**
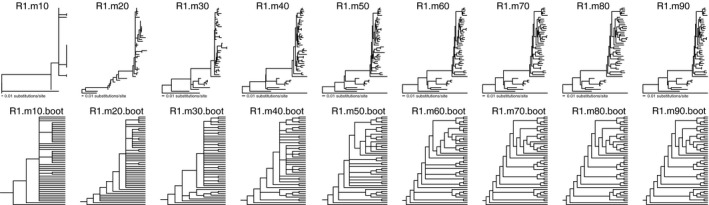
Variation among tree topologies and resolution (top row, phylograms; bottom row, cladograms) as a function of the missing data threshold. All trees were derived from the same combination of Stacks parameters (i.e., Run 1 in Table 2). Minimum missing data threshold ranges from 10% (far left) to 90% (far right). Top row depicts tree shape of ML Tree derived from specified analysis; bottom row depicts 50% majority‐rule consensus tree derived from 100 ML bootstrap replicates, with nodes appearing in fewer than 50% of the trees collapsed. Data indicate that very high levels of missing data (i.e., 90%) yield highly resolved and strongly supported topologies

RADseq data yielded a fully resolved and nearly fully supported phylogenetic hypothesis of relationships among species and individuals of *Petalidium* (Figure 7). Analyses demonstrated that higher allowances of missing data increased both phylogenetic resolution and node support (Figure 8). One of our best‐supported and most fully resolved trees (e.g., R1.m90, corresponding to Run 1 with 90% missing data threshold; Table 2) yielded ten distinct clades plus four additional single‐accession lineages (*P. oblongifolium*, *P. aromaticum*, *P. cirrhiferum*, and *P*. sp. 8 [cf. *variabile*]) comprising the *Petalidium* radiation (Figure 7). Of the 45 total nodes present in our most fully resolved phylogenies such as that of R1.m90, 42 were supported by ML bootstrap values ≥70% (Figure 7). The only unsupported nodes in the R1.m90 phylogenetic hypothesis were those pertaining to the monophyly of Clades 6–10, the sister group relationship between *P. coccineum* and *P. bracteatum*, and the sister group relationship between two of three accessions of *P. engleranum* (Figure 7). The phylogenetic hypothesis derived from assembly to the reference genome gave similar relationships albeit less supported and less resolved than the phylogenetic hypothesis of the de novo‐assembled data that yielded R1.m90 (Figure 7). Of the 11 species for which multiple accessions were included in phylogenetic analyses, all were monophyletic (Figure 7). RADseq data used in this study successfully resolved both species limits and species‐level relationships.

### 3.5 Divergence timing

**Figure 9:**
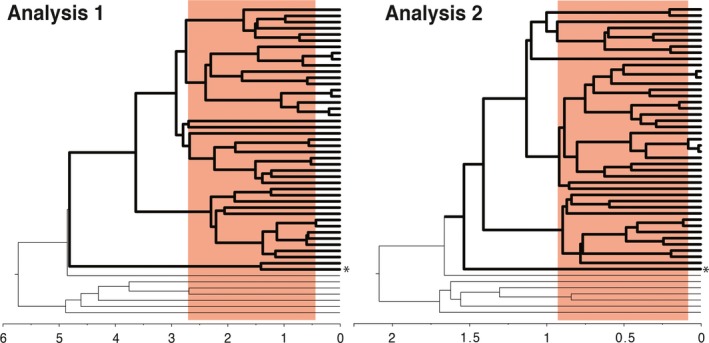
Schematic depicting estimated divergence times of stem, crown, and core *Petalidium* based on results of Analysis 1 (left) and Analysis 2 (right). Both analyses yield ages consistent with a recent and rapid radiation of species in the ultra‐arid deserts of Namibia, Angola, and areas immediately adjacent. Bold branches represent species of *Petalidium*; outgroups depicted by thinner branches. Asterisk represents placement of the only non‐African member of *Petalidium*, *P. barlerioides*. *X*‐axis in millions of years

Overall, both analyses of Sanger sequence data yielded extremely poor node support as assessed by posterior probabilities: Among African *Petalidium*, only one node in both Analysis 1 and Analysis 2 was represented by a posterior probability ≥95% (see Supporting Information as well as trees in DRYAD). Regarding the root of *Petalidium*, Analysis 1 resolved *P. canescens* + *P. barlerioides* as the earliest diverging within the genus, but this sister group relationship was not supported (posterior probability = 0.715; Supporting Information). In contrast, Analysis 2 resolved only *P. barlerioides* as the earliest diverging species (posterior probability = 1.0) and sister to all African species (posterior probability = 1.0; Supporting Information); this relationship represents only the second supported node across either topology. Both analyses yielded relatively young age estimates for the origin and subsequent diversification of *Petalidium,* albeit on slightly different temporal scales (Figure 9). Whereas Analysis 1 indicated stem and crown ages of *Petalidium* of ~4.8 Ma, Analysis 2 indicated stem and crown ages several million years younger: 1.7 and 1.5 Ma, respectively. According to analysis 2, the diversification of all African species began ~1.4 mya while according to Analysis 1, the diversification of all African species but one began ~3.6 mya (Table 3; see Supporting Information for credibility intervals).

## 4 DISCUSSION

Phylogenetic analyses based on RADseq data fully resolved a first estimate of evolutionary relationships within the enigmatically diverse angiosperm genus *Petalidium*. These data together with data from earlier studies further demonstrate the power of RADseq data for analyses at the population as well as species levels. Phylogenetic relationships were successfully and strongly reconstructed in de novo data assemblies with very high thresholds (e.g., ≥90%) of missing data. Our comparison of de novo assemblies to an assembly generated using a reference genome showed that de novo assemblies are capable of generating phylogenies with better resolution and node support, particularly when loci with higher thresholds of missing data (e.g., ≥60%) are included in the analysis. The improved phylogenetic performance of de novo assemblies and those that include loci with high thresholds of missing data derives from the greatly increased number of SNPs that are employed in downstream phylogenetic analyses.

### 4.1 Phylogeny and evolution of petalidium

Attempts to reconstruct relationships among *Petalidium* using Sanger‐based approaches have yielded trees that are nearly void of node support and full of polytomies (Supporting Information). Although the primary purpose of the present study was not to explicitly compare Sanger‐based phylogenies to those of NGS phylogenies given that such differences in performance have been demonstrated clearly by numerous recent works (e.g., Cruad et al., 2014; Massatti, Reznicek, & Knowles, 2016; Nicholls et al., 2015), visual comparison of bootstrap support in Figure 7 (derived from NGS data) to support in divergence time trees in the Supporting Information (derived from Sanger data) clearly depict much poorer performance of the Sanger sequence data.

Regardless of data type (RADseq vs. Sanger), one clear result from all analyses in our study is that *Petalidium* forms a monophyletic group. Strong morphological synapomorphies exist to confirm this monophyly (see Tripp et al., 2013). The single most salient feature of species of *Petalidium* is the paired, leaf‐like bracts, those typically large and prominent, that enclose flowers (seen readily in Figure 1a,c,k,l,o,w,x,y,z,aa,bb). Additional synapomorphies include two‐seeded capsules with elastically dehiscent septa that break away from capsule walls at maturity (in addition to explosive capsule dehiscence typical of all Acanthaceae s.s.) and pollen that is ellipsoid, triangular in polar view, 12‐pseudocolpate, and triporate, with each pore being flanked by two sexine lips along the equatorial axis plus two additional areas of raised tectum along the polar axis (Tripp et al., 2013).

Prior to the present study, there have been two major attempts at treating relationships within the genus in any level of detail: the works by Obermeijer (1936) and Meyer (1968). Both proposed infrageneric classifications consisting primarily of two sections (a third monotypic section was also proposed in Obermeijer 1936): one containing the species with regular, five‐parted calyces (highlighted in red in Figure 7), and the other containing species with irregular, four‐parted calyces (highlighted in blue in Figure 7); neither *P. oblongifolium* nor *P. aromaticum* were treated in infrageneric classifications in Obermeijer (1936) or Meyer (1968). Our phylogenetic results corroborate this division. Although our RADseq sampling did not include the only species of *Petalidium* found outside of Africa*, P. barlerioides*, our Sanger sequence phylogeny derived from Analysis 2 shows this species to be strongly supported as sister to all African species and we predict that RADseq data will in the future similarly resolve this relationship. Regarding calyx arrangement of unplaced species: (1) *Petalidium barlerioides* has a five‐parted calyx like the red group in Figure 7; (2) we have been unable to study the type specimen of *P. oblongifolium* to deduce its configuration, which we predict will be five‐parted based on Figure 7; and (3) *P. aromaticum* has a four‐parted calyx and thus should be treated among species highlighted in blue in Figure 7 in forthcoming taxonomic works (E. Tripp & K. Dexter, in prep.).

Among the African species, our topology in Figure 7 is corroborated by additional aspects of plant morphology. First, clade 2 is notable in uniting *P. luteo‐album* and *giessii*, two species very similar in their large yellow flowers with cordate bracts that almost completely encompass the flower and in possessing floral fragrance. The latter character is otherwise largely absent in Acanthaceae. Second, clades 3, 4, and 5 are composed primarily of species with marked, compact, head‐like inflorescence structures (Figure 1o–t), with the exception of *P. engleranum* (Figure 1u), which does not share this inflorescence structure. Thus, it can be hypothesized that this distinctive morphology evolved relatively early in the history of *Petalidium*, characterizes three different clades that together do not form a clade with respect to remaining *Petalidium*, and that there have been reversals in the *P. engleranum* lineage and in the large clade comprising clades 6–10 and *P. cirrhiferum* (alternatively, this morphology has been gained three times: once each in clades 3, 4, and 5). Third, clade 3 contains two species that are distinct in their minute, burgundy‐colored inflorescences that are likely pollinated by long‐tongued flies. Fourth, clade 10 is composed entirely of species with large, red, tubular, apparently sunbird‐pollinated flowers, which are not found in the genus outside of this clade. Lastly, clades 6 through 10 (Figure 7) are almost entirely restricted to and emblematic of Namib Desert (primarily, Kaokoveld) landscapes (Figure 1b,d,e,g,h). We suspect that the eight species of *Petalidium* that are endemic to Angola and that we have not sampled will also fall into this clade. This clade therefore likely represents the bulk of diversification in the genus.

Remarkably, all species for which multiple samples were included in our analyses formed reciprocally monophyletic groups, which is particularly surprising for *P. variabile* that shows substantial morphological heterogeneity (e.g., see Figure 1 for flower color variation). The repeated monophyly of species suggests a relatively rapid process of lineage sorting, little gene flow among the sampled taxa, or that we have not yet included other taxa or individuals that are likely to be “problematic” (e.g., putative hybrid plants that we have collected in the field). The present phylogenetic framework establishes an important resource for reconstructing a fuller phylogenetic and evolutionary story of *Petalidium*.

### 4.2 Divergence timing in Petalidium

Our crown ages for *Petalidium* indicate a relatively young age of origin, ranging from 4.8 to 1.5 mya depending on the primary fossil calibration. These age estimates include *P. barlerioides*, hypothesized to be sister to the African radiation (and supported by Analysis 2; Supporting Information), and if they approximate true divergence times, then the crown age of the African clade of *Petalidium* dates to ca. 3.6 to 1.4 my a (see Supporting Information for credibility intervals). Using the approach of Magallón and Sanderson (2000), these crown ages yield an estimated net diversification rate ranging from 0.8 to 2.1 species per my, which is on par with rates found for diverse lineages in tropical, Mediterranean, and alpine ecosystems (Madriñan, Cortés, & Richardson, 2013; Valente, Savolainen, & Vargas, 2010). Thus, despite life in an extreme and unpredictable environment, species of *Petalidium* are diversifying rapidly in this region. The above analyses are consistent with a hypothesis of active radiation of the genus, primarily in Namibia and Angola. Of interest is fossil midden evidence that indicates *Petalidium* has dramatically increased in abundance over at least the last 1,000 years, coincident with the continued drying of the environment in this region (Gil‐Romera et al., 2006).

### 4.3 RADseq data usage

#### 4.3.1 Strategies and Impacts

Numerous studies have highlighted various benefits of reference‐based assembly (Andrews et al., 2016; Davey & Blaxter, 2010; Gonen et al., 2015; Hipp et al., 2014; Rubin et al., 2012). McCluskey and Postlethwait (2015) in particular harnessed the power of a reference genome to improve quality filtering of reads by removing those that mapped to repeat regions, mapped to multiple regions of a genome, or mapped with low support. We agree that reference‐based methods yield data that are generally higher in quality than are data derived from de novo assembly. However, results from the present study together with prior studies to have compared reference‐based to de novo read assembly (McCluskey & Postlethwait, 2015; Stetter & Schmid, 2017) demonstrate that reference‐based approaches result in the dropping out of considerable numbers of RADseq loci. These results highlight the trade‐off in using reference‐based methods in that they yield a higher quality (not explicitly addressed in the present study, but see McCluskey & Postlethwait, 2015) but a lower quantity of RADseq loci. In our study, only at the lowest thresholds of missing data (<30%) did a reference‐based assembly outperform the de novo assemblies in our study (Figure 6). Moreover, topologies resulting from our reference‐based assembly had overall fewer well‐supported nodes, reflecting the fewer numbers of SNPs recovered with this assembly method (Figure 6). This raises a question of how much impact the evolutionary relatedness of the reference genome has on assembly and thus phylogenetic resolution and support. Stetter and Schmid (2017) compared read mapping to a closely related and a more distantly related plant genome and found dramatic variation in the number of aligned reads (75% compared to 26%). In this study, an average of 30% of reads were mapped to the reference genome, which likely relates to relatively high phylogenetic divergence of our reference genome compared to the ingroup (crown age of *Ruellia ~*10.6 Ma compared to the much younger crown age of *Petalidium* [4.8–1.5 Ma]). This effect may be ameliorated by choosing a different read mapper (vs. Bowtie as used in this study) that is more optimized for mapping to a divergent genome (e.g., Stampy; Lunter & Goodson, 2011). Future studies to compare assemblies to reference genomes of varying degrees of phylogenetic relatedness will provide further insight as to whether and when to employ a reference genome, which will likely depend on phylogenetic distance to the ingroup and need to balance high quality with high quantity of loci (i.e., the trade‐off). Future research could also explore the effects of mapping reads to multiple reference genomes simultaneously, which while more computationally intensive, could yield improvements in numbers of called loci as well as improve confidence in the orthology of resulting loci. Another strategy would be to first conduct a reference‐based assembly, then a de novo assembly on the remaining reads thereby preserving positional information for at least part of the dataset.

For over two decades, the problem of missing data has been and remains central to discussion of phylogenetic reconstruction (Grievnik, Penny, & Holland, 2013; Jiang, Chen, Wang, Li, & Wiens, 2014; Maddison, 1993; Roure, Baurain, & Philippe, 2013; Simmons, 2014; Wiens, 1998). In 2003, Wiens published a landmark paper in which he used simulations to demonstrate that reduced phylogenetic accuracy commonly associated with missing data actually reflects a dataset that lacks enough information rather than having too much missing data per se. Wiens (2003) further discussed the potential for accurate phylogenetic placement of taxa with extremely high levels of missing data. In our study, after building matrices that included loci with very high thresholds of missing data across samples (e.g., 90%), even accessions represented by very few SNPs (e.g., R1.m90, *Petalidium giessii*‐3: 2,502 of 53,972 SNPs or 5%) were placed with high bootstrap support (see Figure 7). From our and numerous other recent studies that have employed RADseq data (e.g., Rubin et al., 2012; Wagner et al., 2013; Wessinger et al., 2016), loci with much missing data are better thought of as beneficial rather than detrimental. Considering the balance between signal and noise, our analyses demonstrate that these loci add more signal than noise and should thus be included.

In this study, we evaluated the performance of different approaches to data assembly based on support levels in phylogenetic hypotheses that were derived from them. Ideally, we would know the true phylogeny and be able to evaluate performance based on the accuracy of the phylogenetic hypotheses derived from different approaches, but as with most nonexperimental studies of extant taxa, the true phylogeny is unknowable (although simulation study can be helpful). Our analyses do show a clear monotonic relationship between the number of SNPs present in a given data assembly and the level of support in the phylogeny derived from the data assembly (Figure 6). There are good a priori reasons to believe that more SNPs will result in not only better‐supported phylogenies, but also more accurate phylogenies (see Wortley, Rudall, Harris, & Scotland, 2005; Xi, Liu, & Davis, 2015). Another important point in this context is that phylogenetic hypotheses derived from concatenated sequence data from many loci often result in higher node support values than hypotheses derived from a gene tree–species tree approach (Lambert, Reeder, & Wiens, 2015; Nicholls et al., 2015; Thiergart, Landan, & Martin, 2014). However, the manner in which RADseq data are assembled precludes the straightforward implementation of gene tree–species tree approaches, at least given current computational approaches. In any case, our focus here is on the relative support levels in phylogenies derived from different data assembly approaches, and their relative performances may be the same regardless of whether concatenated or gene tree–species tree phylogenetic analyses are conducted.

Finally, there continues to exist debate about the evolutionary “depth” at which RADseq can be used (Eaton, 2014; Rubin et al., 2012). Assuming some degree of constancy and heritability of rates of molecular evolution at restriction sites, the most important variables that are likely to impact the efficacy of RADseq are the relative time span of species diversification in the focal lineage and how long ago it occurred. Older diversification inherently correlates with fewer loci because of mutation accumulation at restriction sites and because of greater difficulty in assessing sequence orthology during loci identification. Indeed, Rubin et al. (2012) demonstrated a decrease in the number of orthologous restriction sites with increasing degrees of evolutionary divergence, suggesting that caveats that have been described for using RADseq data across greater time spans should be considered carefully. However, Eaton, Spriggs, Park, and Donoghue (2016) demonstrated high efficacy of RADseq loci in reconstructing deep evolutionary bifurcations as a function of quartet informativeness and redundancy. Numerous studies have successfully used RADseq data to resolve species relationships across substantial evolutionary time spans. Although not all of these explicitly placed phylogenetic results in a temporal framework, a general impression can be garnered from the following macroevolutionary RADseq studies: (1) Wagner et al. (2013) resolved relationships among 16 species of Lake Victoria Cichlids that are likely <15,000 years old; (2) Wessinger et al. (2016) resolved relationships among 75 species of *Penstemon* that have likely diverged since the late Neogene (±2.5 mya); (3) Hou et al. (2015) resolved relationships among five species of *Diapensia* that likely date from the late Miocene (±5.3 mya) to the present; (4) Cavender‐Bares et al. (2015) resolved relationships among seven species of American live oaks that diverged from one another over an approximately 7 my time period; (5) Cruad et al. (2014) resolved relationships among 18 species of ground beetles with divergences up to 17 mya; (6) Hipp et al. (2014) resolved relationships among 28 species of American oaks, a clade that diversified from 23–33 mya to the present but primarily over a 20 my time span (i.e., from 5–25 mya; unpublished data courtesy of P. Manos); (7) Rubin et al. (2012) and Cariou, Duret, and Charlat (2013) resolved relationships among 12 species of *Drosophila*, whose genomes were approximately five to 63 my divergent; and (8) Herrera and Shank (2016) resolved relationships among 12 species of deep sea corals, with maximum divergences of at least 80 my. In our study, crown *Petalidium* evolved between 4.8 and 1.5 mya, with the diversification of 38–39 African species beginning 3.6 to 1.4 mya. From the above, it seems clear that RADseq data are remarkably robust to varying degrees of evolutionary divergence and that obtaining sufficient numbers of SNPs is the single biggest predictor of capacity to resolve a supported phylogeny.

## 5 CONCLUSION

This investigation used RADseq data to investigate a radiation of plants in an ultra‐arid ecosystem and provided a first phylogenetic hypothesis for evolutionary relationships among *Petalidium*. Results add further support to the growing consensus that these data are exceptionally useful at numerous phylogenetic scales. Phylogenetic relationships among *Petalidium* herein resolved will contribute meaningfully to future studies that investigate correlates of speciation in the genus, particularly with respect to factors that have promoted high infrageneric diversification in a geographically limited area, which defies general patterns of plant diversity in deserts worldwide.

## AUTHOR CONTRIBUTIONS

EAT, YET, and KGD conceptualized the study; EAT and KGD conducted the fieldwork; EAT and YET designed and executed the experiment; YET conducted the analyses; YZ assembled and annotated genome for *Ruellia speciosa;* EAT, YET, and KGD wrote the manuscript.

## CONFLICT OF INTEREST

None declared.

## DATA ACCESSIBILITY

Data associated with this study have been deposited into GenBank (Study #PRJNA392452; SRA #SRP110762) and Dryad (https://doi.org/10.5061/dryad.1t1f5).
