# Supplementary figures and images for: RADseq dataset with 90% missing data fully resolves recent radiation of Petalidium (Acanthaceae) in the ultra‐arid deserts of Namibia
Source: Ecol Evol. 2017 Aug 30;7(19):7920–36. doi: 10.1002/ece3.3274 (PMC5632676; doi:10.1002/ece3.3274)

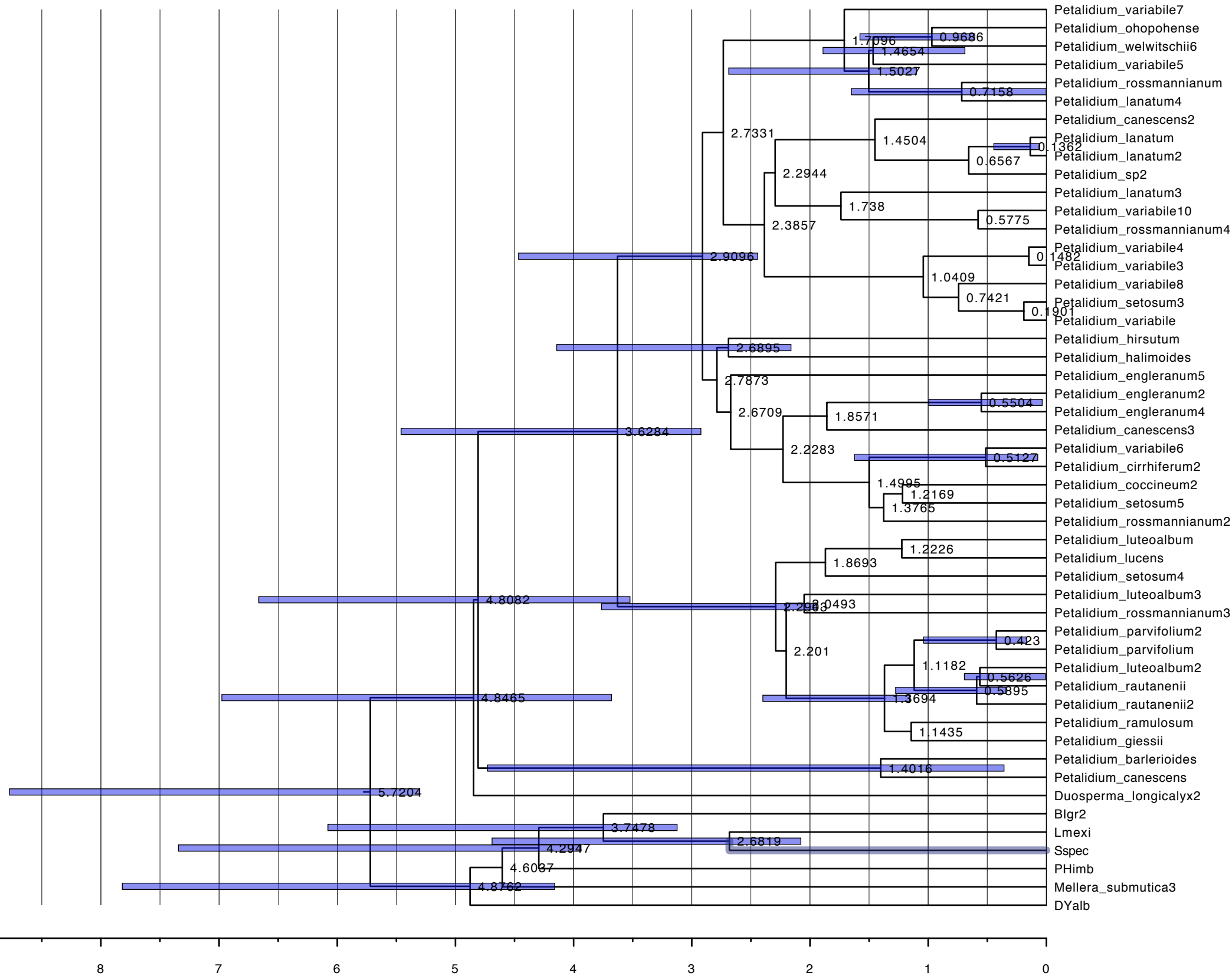

Supplement: Supplementary file 1 [file ECE3-7-7920-s001.pdf]

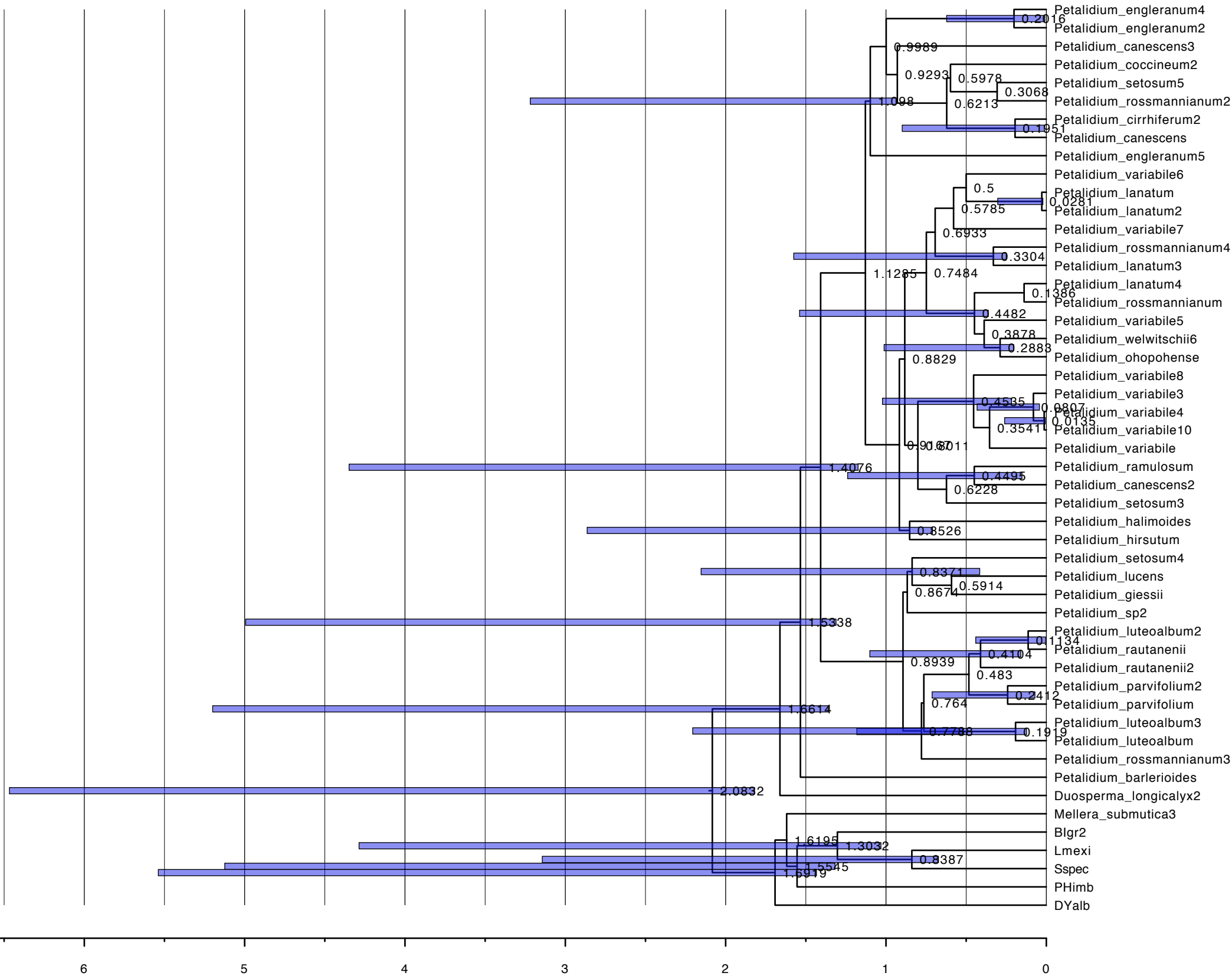

Supplement: Supplementary file 2 [file ECE3-7-7920-s002.pdf]

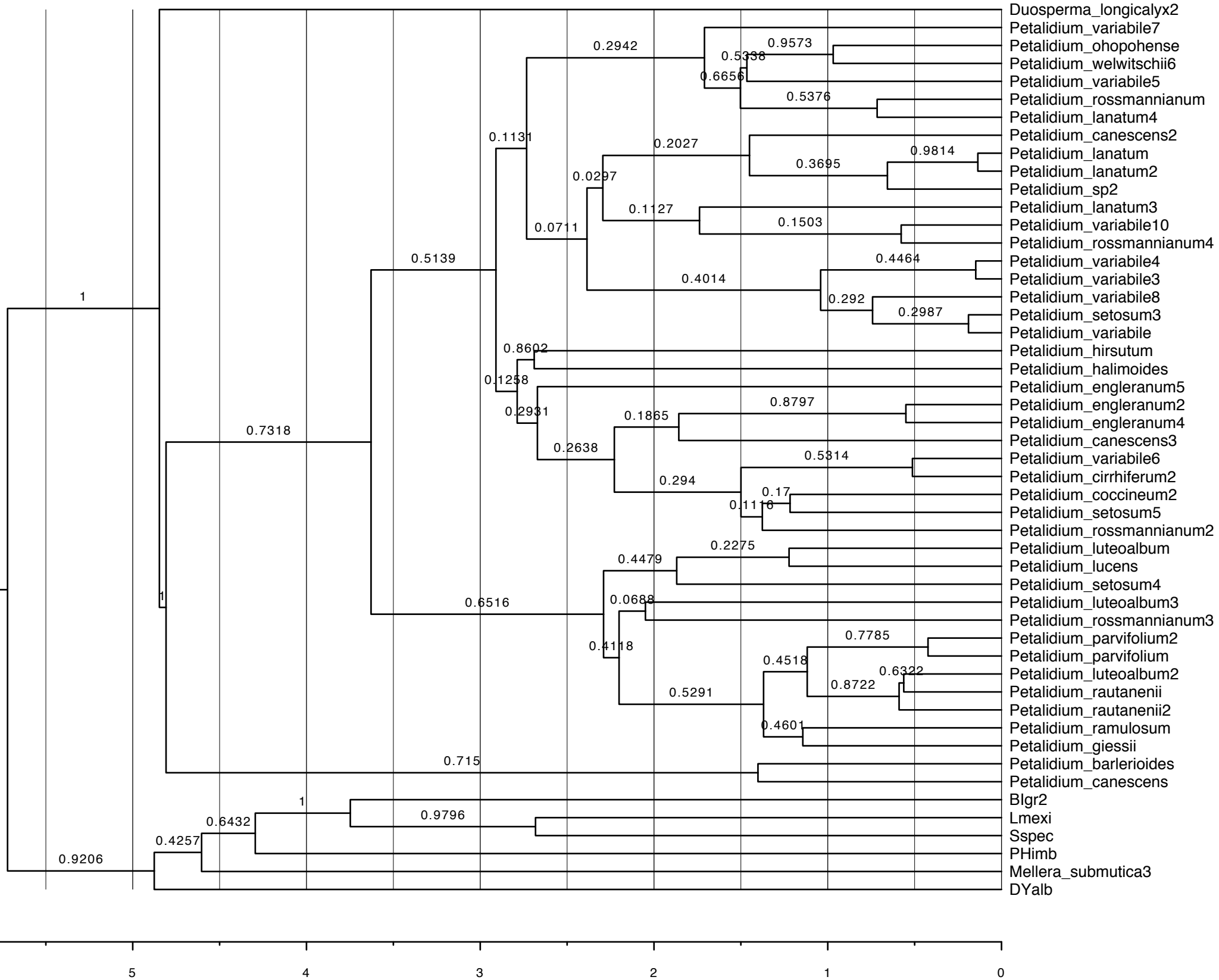

Supplement: Supplementary file 3 [file ECE3-7-7920-s003.pdf]

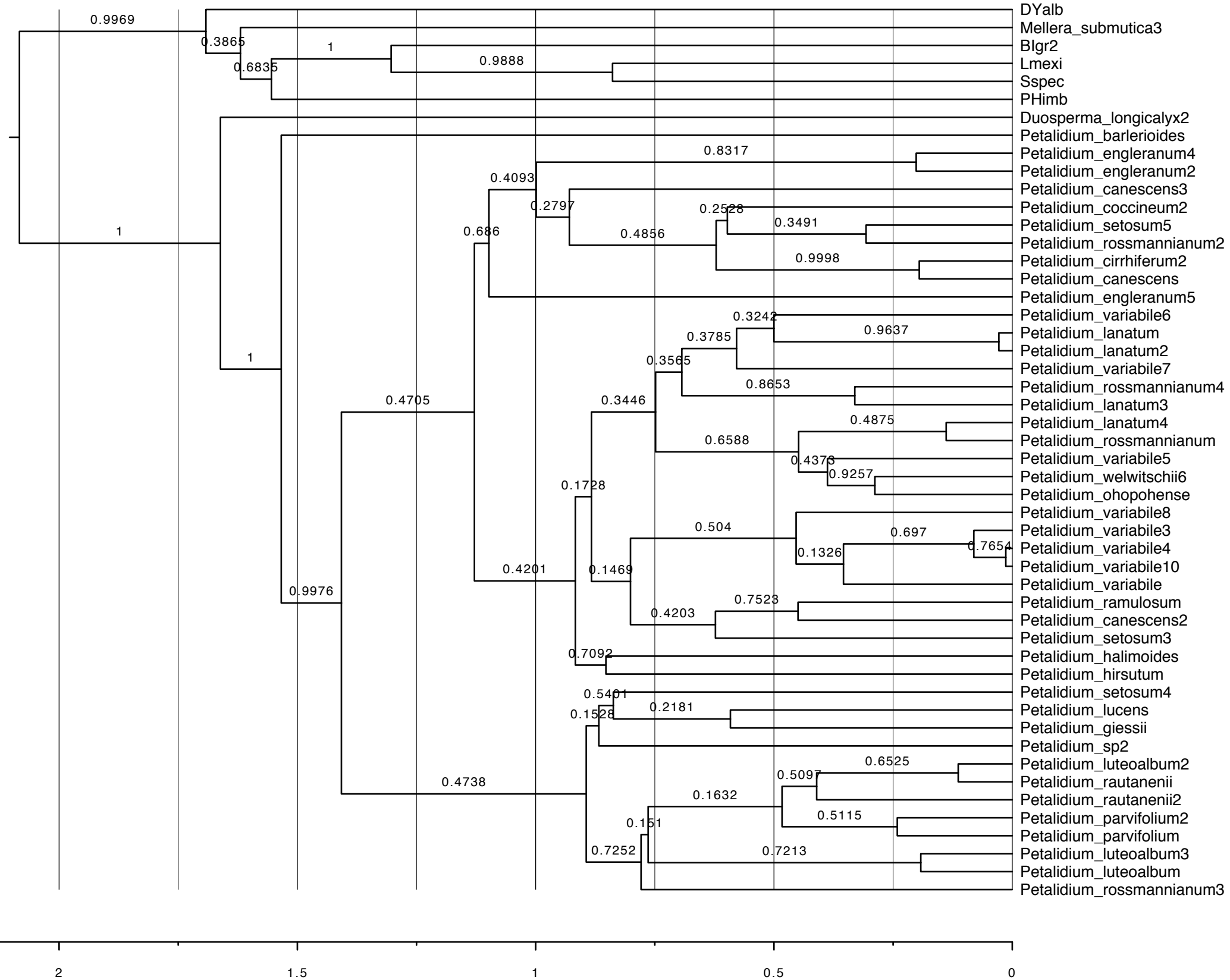

Supplement: Supplementary file 4 [file ECE3-7-7920-s004.pdf]
