# Supplementary material for: RADseq dataset with 90% missing data fully resolves recent radiation of Petalidium (Acanthaceae) in the ultra‐arid deserts of Namibia
Source: Ecol Evol. 2017 Aug 30;7(19):7920–36. doi: 10.1002/ece3.3274 (PMC5632676; doi:10.1002/ece3.3274)
Supplement: Supplementary file 5 [file ECE3-7-7920-s005.pdf]

Yi-Hsin Erica Tsai
(yihsin.erica.tsai@gmail.com)
University of Colorado Boulder
Updated 6/6/2016

GBS with size selection lab protocol*

*Adapted and modified from: Parchman TL, et al. (2012) Genome-wide association genetics of an adaptive trait in lodgepole pine. Mol. Ecol. 21:2991-3005.

Double Restriction Digestion

1. Prepare Master Mix I (125% of what you will need).
2. Distribute 3 ul of Master Mix I to each well.
3. Add 6 ul of sample DNA to each well (20-150 ng/ul) (120-900ng total).
4. Cover, seal, mix gently, and centrifuge.
5. Total reaction volume = 9 ul. Incubate at 37°C for 15[i] minutes, followed by 65°C for 45 minutes, hold at 4°C. Use program “2XDIGEST”[ii].

Master Mix I

**Reagent**	**1X**	**30X (24 samples)**	**60X (48 samples)**
H2O	0.82	24.6	49.2
10X T4 Buffer	1.15	34.5	69
1M NaCl	0.60	18	36
BSA (20mg/ml)	0.03	0.9	1.8
EcoRI-HF (20,000u/ml)	0.28	8.4	16.8
MseI (10,000u/ml)	0.12	3.6	7.2
**Total**	**3 ul**	**90ul**	**180ul**

Adaptor Ligation

1. Prepare MseI and EcoRI adaptors
    a. IDT has annealed them for us already
    b. 1uM EcoRI adaptors working solutions thawed
        i. 10uL of 10uM stock in 90uL H2O.
        ii. Note: there are 100uM and 10uM stocks, READ THE LABELS carefully.
    c. 10uM MseI working solution thawed
        i. 20uL of 100uM stock in 180uL H2O.
2. Prepare Master Mix II (125% of what you will need).

Master Mix II

**Reagent**	**1X**	**30X**	**60X**
H2O[iii]	0.072	2.2	4.3
10X T4 Buffer	0.1	3	6
1M NaCl	0.05	1.5	3
1 mg/mL BSA	0.05	1.5	3
MseI Adaptors (10uM)	1	30	60
T4 DNA Ligase	0.1675	5.0	10.0
**Total**	**1.4 uL**	**43.2 ul**	**86.4 ul**

3. Add 1.4 ul of Master Mix II to each restriction-digested reaction. Keep MMII on ice until use.
4. Add 1 ul of EcoRI adaptors (1uM) to each well (MseI adaptors are already in MMII). See adaptor table (Appendix 1) for barcode key.
5. Total reaction volume is 11.4 ul. Cover, seal, gently mix, and centrifuge.

6. Incubate at 16^oC overnight, heat inactivate at 65 ^oC for 10 minutes, hold at 4^oC. Use program "LIGATE02".
7. Dilute the restriction-ligation reaction with *89 ul* of 0.1X TE. Store at 4^oC for up to one month.

PCR Amplification

"To ameliorate stochastic differences in PCR production of fragments in reactions, we run two separate 20uL reactions per restriction-ligation product, and later combine them."[iv]

1. Prepare IllPCR primers.
    a. Mix 30uL Illpcr1, 30uL Illpcr2 (of 100uM stock) with 540uL H2O (makes 5uM working solution of each oligo).
2. Prepare PCR Master Mix (125% of what you will need). Remember we are preparing 2X PCR reactions.

PCR Master Mix

**Reagent**	**1X**	**60X**	**120X**
H_2O	7.3	438	876
5X Phusion HF Buffer	4	240	480
IllPCR primer mix (5uM)	2	120	240
2.5 mM dNTPs	1.6	96	192
Phusion DNA Polymerase	0.1	6	12
**Total**	**15uL**	**900**	**1900**

3. Add 15 ul of PCR Master Mix into new plates or tubes. Keep on ice until use.
4. Vortex and centrifuge the diluted restriction-ligation product. Add **5**[v] **ul of product** to the appropriate tube or well. 20uL total volume.
5. Thermalcycle using program "ILLPCR00_Hotstart" (98^oC Hot Start; 98^oC for 60s; 30 cycles of 98^oC for 20s, 60^oC for 30s, 72^oC for 40s; 72^oC for 10m; 4^oC hold).

Gel Purification ***

*** An alternative to doing the size selection via gel is to pool the PCR products and run an AMPure bead size selection and cleanup. This might be very economical (~$15 per tube) depending on the pooling. I'd look into this seriously next time. Not sure if this bead cleanup step could replace the size selection step entirely, or if it just fixes the adaptor dimer and other problems that can arise after gel extraction.

1. Pool all samples into a few microcentrifuge tubes as possible. (For 2 plates, divide into 6-10 tubes).
2. Evapoconcentrate the PCR product (vacuum centrifuge for ~30-45[vi] min).
3. Run the PCR product out on a 2% agarose gel at 100 volts for 2.5 hours.
    a. Use 50% high resolution agarose, 50% normal agarose.
    b. Use 1X TAE with 1X SYBR Safe.
    c. Make sure you use THIN combs with wide lanes.
    d. Pour gel thick to fit more in a lane.
    e. Make sure the gel is fully solid before loading. Load the gel dry.
    f. Include 20 ul of 50 bp ladder at multiple spots on the gel, leaving 1 lane between ladder and samples. Make sure you load enough of the ladder to fully cover the lane. Add 20uL loading dye per wide lane.
    g. Load 60-80 ul of pooled samples per well without loading dye.
    h. Add just enough buffer to just go up the sides of the gel, run for 10-15 minutes, then add more buffer before continuing.[vii]
4. Cut the region of gel between 300-400 bp[viii]. Be sure to cut off excess gel (anything that doesn't light up), otherwise you will be doing a ridiculous amount of purifications.
5. Purify the excised gel using the Qiaquick gel purification kit with the following protocol that closely follows the Qiagen instructions:
    a. Turn on the water bath to 50^oC.

b. Weigh 50mL Falcon tubes before addition of gel slices.
c. Place <8g of gel in each Falcon tube, record the weight. Use the gel weight to calculate how many spin columns to use per Falcon tube. Each spin column can handle <400mg of gel.
d. Add up to 6 volumes of Buffer QG to the Falcon tubes (basically to the 40mL mark).
e. Incubate at 50°C for 10 min (or until the gel slice has completely dissolved). Vortex every 2-3 minutes to help dissolve. If it can't dissolve, add more buffer (may need t split into more Falcon tubes).
f. Add 1 gel volume of isopropanol (basically to the 50mL mark).
g. Place the calculated number of QIAquick spin columns in the provided 2 ml collection tube in a rack. To bind DNA, apply 750uL of the sample to each of the QIAquick columns and centrifuge for 1 min. Discard flow-through and place spin columns back into the collection tubes.
    i. Repeat applying 750uL of sample to each column, centrifuging, and discarding the flow through until all of the sample in the Falcon tube has been applied. Try to evenly distribute the sample across its set of spin columns.
h. Add 0.5 ml Buffer QG to the QIAquick column and centrifuge for 1 min.
i. Discard flow-through and place the QIAquick column back into the same tube.
j. To wash, add 750 ul Buffer PE to the QIAquick column, let the column stand for 5 min, and centrifuge for 1 min.
k. Centrifuge the QIAquick column once more in the provided 2ml collection tube for 5 min at 17,9000 x g (13,000 rpm) to remove residual wash buffer. Be sure to remove all of the remaining ethanol.
l. Place QIAquick column into a clean 1.5 ml microcentrifuge tube.
m. To elute DNA, add 40 ul water[ix] to the center of the QIAquick membrane, let stand for 10 min, centrifuge column for 1 min.
n. Once the samples are centrifuged, combine all samples into a single 1.5 ml tube.
o. The salts from buffer QG make a huge mess. So be sure to budget time for clean up.

6. You may need to concentrate the product using the speedvac or a column elution method as recommended by the sequencing facility.
7. Check concentration via nanodrop before submission to the sequencing facility.

Appendix 1: Barcode key

Names of barcodes and their locations on the plate

H	G	F	E	D	C	B	A	
GBS386	GBS299	GBS274	GBS213	GBS189	GBS168	GBS113	GBS89	1
GBS1096	GBS893	GBS790	GBS763	GBS628	GBS514	GBS480	GBS448	2
GBS110	GBS99	GBS95	GBS65	GBS47	GBS36	GBS1186	GBS1136	3
GBS516	GBS430	GBS424	GBS399	GBS358	GBS272	GBS174	GBS145	4
GBS1201	GBS1132	GBS831	GBS684	GBS676	GBS673	GBS653	GBS646	5
GBS262	GBS248	GBS194	GBS161	GBS157	GBS7	GBS1251	GBS1240	6
GBS527	GBS444	GBS392	GBS332	GBS329	GBS305	GBS282	GBS278	7
GBS1123	GBS915	GBS901	GBS792	GBS761	GBS688	GBS648	GBS569	8
GBS737	GBS693	GBS683	GBS610	GBS468	GBS279	GBS1281	GBS1167	9
GBS975	GBS941	GBS913	GBS902	GBS881	GBS880	GBS837	GBS785	10
GBS822	GBS793	GBS773	GBS553	GBS356	GBS239	GBS147	GBS1222	11
GBS1159	GBS1135	GBS1015	GBS952	GBS943	GBS904	GBS887	GBS875	12

Barcode sequences (sense strand) and their locations on the plate

H	G	F	E	D	C	B	A	
ATAGGCG	AGCAAGG	AGACCGC	ACGGTCT	ACGAATC	ACCGCAG	AATATAC	AAGTAAG	1
TCAGAAC	GCATTGC	CTCGATT	CTATGAT	CCTACGC	CAGCTCG	CAAGAGG	ATTAGTT	2
AATACTCT	AATAACTC	AAGTTCGG	AAGAGATT	AACTCGGT	AACTAACG	TGATTCT	TCGCCTG	3
CAGCTTCG	ATGATGGC	ATGAATAG	ATATCTGC	ATAAGCCT	AGACCAGT	ACCGTTAG	AATTGCAT	4
TGCGACGT	TCGAGCTG	CTTCTATG	CGCAGGAG	CGATTGAT	CGATCATG	CCTTCCTC	CCTGAAGG	5
AGAACTAGT	ACTGCATCT	ACGAGCGTA	ACCATATAT	ACCAGGCCG	AACCATGGA	TTCAGGTT	TTAGGTAC	6
CAGTTGACG	ATGGTCCTA	ATATAGCAG	AGGTAGAGT	AGGCTGGCG	AGCCGCAGT	AGAGAATTG	AGACGGTTC	7
TCCGTCGAA	GCGTCCAGG	GCCGTAGGT	CTCGCCGAT	CTATATGCT	CGCCAAGGC	CCTGGAACG	CCAAGCCGG	8
CGTTATAACG	CGCCTGATAG	CGCAGAGACG	CCGCCTATGG	CAACCTTAAG	AGACGTTAGG	TTGGACGAG	TGAACCTTC	9
GGTTCCAATG	GGATAAGAGG	GCGGTTACTG	GCCTAGTAAG	GATGGAATAG	GATGCTGACG	CTTGGCATTG	CTCCGTTATG	10
CTTACCATCT	CTCGCGCAGT	CTCATAGATT	CATGGCAACT	ATAAGAAGGT	ACTCCTAGGT	AATTGGAATT	TGGCTGAACG	11
TCTCTTCATT	TCGCCGTAGT	GTTCATATAT	GGCCGGTATT	GGATTGCAGT	GCCTGGAAGT	GCAGGTCAGT	GATCGTAGTT	12

Endnotes

---

[i] Shortened the incubation time to just 15 minutes. I think we may have overdigested the DNA previously. If you're doing this for the first time, I'd repeat the digestion and PCR several times and check it on a gel to make sure the digestion time is optimal.
[ii] Any incubation time >15min should work, followed by inactivation time >20min should work, because we are using fast acting enzymes. Also, we shouldn't let it go overnight due to increased star activity.
[iii] These volumes are a bit strange, but they match previous protocols.
[iv] From Parchman et al.'s protocol.
[v] The amount "5uL" is by design to make it easier to pipette via a multichannel pipette.
[vi] This step always takes forever. Can make things go faster by distributing liquid among many tubes.
[vii] It was unclear whether or not running the gel dry without loading dye worked well. We ended up just adding loading dye, which made the gel running part easier. However, it doubled the amount of gels that needed to be run and thus increased the amount of gel that needed to be cut and extracted.
[viii] Last time due to our overdigested product, we excised the 250-500bp region. If the digestion was optimized first, then we could choose a narrower fragment size range, like 300-400bp.
[ix] Do not elute with EB. It makes things much easier to concentrate via speed-vac if you don't have to worry about excess salts.
