# Supplementary material for: RADseq dataset with 90% missing data fully resolves recent radiation of Petalidium (Acanthaceae) in the ultra‐arid deserts of Namibia
Source: Ecol Evol. 2017 Aug 30;7(19):7920–36. doi: 10.1002/ece3.3274 (PMC5632676; doi:10.1002/ece3.3274)
Supplement: Supplementary file 7 [file ECE3-7-7920-s007.docx]

Supplemental Information.

The supplement to this study contains the following items, each of which is described briefly via captions that follow:

1. Supp Fig_TS1_MCC_3 mil burnin_target_credibility intervals.pdf:

This figure forms the basis of Figure 9 (Analysis 1) in the main document but additionally shows 95% HPD intervals for all branches.

2. Supp Fig_TS2_MCC_5.1 mil burnin_target_credibility intervals.pdf:

This figure forms the basis of Figure 9 (Analysis 2) in the main document but additionally shows 95% HPD intervals for all branches.

3. Supp Fig_TS1_MCC_3 mil burnin_target_posterior probs.pdf

This figure forms the basis of Figure 9 (Analysis 1) in the main document but additionally shows posterior probabilities associated with each branch.

4. Supp Fig_TS2_MCC_5.1 mil burnin_target_posterior probs.pdf

This figure forms the basis of Figure 9 (Analysis 2) in the main document but additionally shows posterior probabilities associated with all branch.

5. Supp Text_GBS_YET protocol.docx:

This document contains the wet laboratory protocol for the GBS experiment.

6. Supp_barcode_info.zip:

This zip file contains multiple files concerning the design and details of the 96 variable length barcodes used for an Illumina single end run. See README.txt within the zip file for more details.

7. Supp_Script_from_rawseq_to_trees.zip:

This zip file contains an example workflow for processing raw RADseq data to the output and summary of phylogenetic trees. See the README.txt within the zip file for more details.
